# Combination of terbium-161 with somatostatin receptor antagonists—a potential paradigm shift for the treatment of neuroendocrine neoplasms

**DOI:** 10.1007/s00259-021-05564-0

**Published:** 2021-10-08

**Authors:** Francesca Borgna, Stephanie Haller, Josep M. Monné Rodriguez, Mihaela Ginj, Pascal V. Grundler, Jan Rijn Zeevaart, Ulli Köster, Roger Schibli, Nicholas P. van der Meulen, Cristina Müller

**Affiliations:** 1 Center for Radiopharmaceutical Sciences, ETH-PSI-USZ, Paul Scherrer Institute, 5232 Villigen-PSI, Switzerland; 2 Laboratory for Animal Model Pathology, Institute of Veterinary Pathology, Vetsuisse Faculty, University of Zurich, 8057 Zurich, Switzerland; 3 The Joint Department of Medical Imaging, University Health Network, 200 Elizabeth St, Toronto, ON M5G 2C4 Canada; 4 South African Nuclear Energy Corporation (Necsa), Pelindaba, Brits, 0240 South Africa; 5 Institut Laue-Langevin, 38042 Grenoble, France; 6 Department of Chemistry and Applied Biosciences, ETH Zurich, 8093 Zurich, Switzerland; 7 Laboratory of Radiochemistry, Paul Scherrer Institute, 5232 Villigen-PSI, Switzerland

**Keywords:** Terbium-161, Auger electrons, NEN, SSTR antagonists, PRRT, Radionuclide therapy

## Abstract

**Purpose:**

The β^¯^-emitting terbium-161 also emits conversion and Auger electrons, which are believed to be effective in killing single cancer cells. Terbium-161 was applied with somatostatin receptor (SSTR) agonists that localize in the cytoplasm (DOTATOC) and cellular nucleus (DOTATOC-NLS) or with a SSTR antagonist that localizes at the cell membrane (DOTA-LM3). The aim was to identify the most favorable peptide/terbium-161 combination for the treatment of neuroendocrine neoplasms (NENs).

**Methods:**

The capability of the ^161^Tb- and ^177^Lu-labeled somatostatin (SST) analogues to reduce viability and survival of SSTR-positive AR42J tumor cells was investigated in vitro. The radiopeptides’ tissue distribution profiles were assessed in tumor-bearing mice. The efficacy of terbium-161 compared to lutetium-177 was investigated in therapy studies in mice using DOTATOC or DOTA-LM3, respectively.

**Results:**

In vitro, [^161^Tb]Tb-DOTA-LM3 was 102-fold more potent than [^177^Lu]Lu-DOTA-LM3; however, ^161^Tb-labeled DOTATOC and DOTATOC-NLS were only 4- to fivefold more effective inhibiting tumor cell viability than their ^177^Lu-labeled counterparts. This result was confirmed in vivo and demonstrated that [^161^Tb]Tb-DOTA-LM3 was significantly more effective in delaying tumor growth than [^177^Lu]Lu-DOTA-LM3, thereby, prolonging survival of the mice. A therapeutic advantage of terbium-161 over lutetium-177 was also manifest when applied with DOTATOC. Since the nuclear localizing sequence (NLS) compromised the in vivo tissue distribution of DOTATOC-NLS, it was not used for therapy.

**Conclusion:**

The use of membrane-localizing DOTA-LM3 was beneficial and profited from the short-ranged electrons emitted by terbium-161. Based on these preclinical data, [^161^Tb]Tb-DOTA-LM3 may outperform the clinically employed [^177^Lu]Lu-DOTATOC for the treatment of patients with NENs.

**Supplementary Information:**

The online version contains supplementary material available at 10.1007/s00259-021-05564-0.

## Introduction

Neuroendocrine neoplasms (NENs) are clinically heterogeneous malignancies, which originate in the neuroendocrine system mostly in the gastro-pancreatic or bronchopulmonary tract [1]. Peptide receptor radionuclide therapy (PRRT) using radiolabeled somatostatin (SST) analogues has been employed since the early 1990s to treat somatostatin receptor (SSTR)-positive NENs [1, 2]. The initially used [^111^In]In-octreotide, a cell-internalizing SSTR agonist, was effective for symptom palliation, but the short tissue penetration of the emitted Auger electrons (< 10 μm) was not sufficient for an effective cancer therapy [3–5]. The application of yttrium-90 was introduced in the late 1990s using the next-generation SSTR agonists, DOTATOC and DOTATATE [6, 7]. Yttrium-90 was more successfully used for PRRT; however, the high energy β¯-particles (Eβ_average_ = 932 keV; max. tissue range: ~ 10 mm) were unfavorable due to the risk of renal damage [8]. The β¯-particle-emitting lutetium-177 (Eβ_average_ = 134 keV, *T*_1/2_ = 6.65 days; max. tissue range: ~ 2 mm [9]) has a more favorable safety profile [8] and is currently the most often employed radiometal for PRRT using DOTATATE (Lutathera™) or DOTATOC [10, 11]. Additionally, the emission of γ-rays makes lutetium-177 useful for γ-scintigraphy and single photon emission computed tomography (SPECT) enabling therapy monitoring and dosimetry [12].

More recently, the challenge of tumor radioresistance triggered the concept of targeted α-therapy, which is particularly effective due to the high linear energy transfer (LET) of α-particles (50–230 keV/μm) [13–15]. PRRT using [^213^Bi]Bi-DOTATOC or [^225^Ac]Ac-DOTATATE in patients, refractory to ^90^Y- and/or ^177^Lu-based PRRT, resulted in partial remission or stable disease [16, 17]. There are, however, a number of uncertainties regarding the application of α-emitters, among those, the unfavorably short half-life of bismuth-213 and the complicated decay scheme of actinium-225, which comprises a considerable risk of undesired side effects. The inability of imaging these α-emitters and, finally, the difficult production scenarios make their application challenging [18–20].

In 2006, Ginj et al. introduced the concept of SSTR antagonists for targeting NENs [21]. Preclinical studies demonstrated much higher tumor accumulation of these non-internalizing SST analogues than for SSTR agonists. In a first-in-human clinical application with four patients with metastatic NENs, the antagonist [^177^Lu]Lu-DOTA-JR11 performed better than [^177^Lu]Lu-DOTATATE [22], which led to a Phase 1/2 multicenter clinical study (NCT02592707). More recently, [^177^Lu]Lu-DOTA-LM3, another SSTR antagonist [23], was tested in 51 patients with metastatic NENs [24]. It resulted in higher tumor doses compared to [^177^Lu]Lu-DOTATOC and was well tolerated in patients [24]. Unfortunately, this promising new class of SSTR antagonists cannot be combined with α-particle emitters with complex decay chain, such as actinium-225, for which effective internalization is an essential prerequisite to avoid toxicity to healthy tissues as a result of released daughter nuclides [18].

Over the last decade, terbium-161 has gained increasing attention as a potential therapy radionuclide [25]. Similar to lutetium-177, it decays by the emission of medium-energy β¯-particles (Eβ_average_ = 154 keV; *T*_1/2_ = 6.95 days [26]) and emits γ-radiation suitable for imaging purposes (Eγ = 48.9 keV, *I* = 17% and 74.6 keV, *I* = 10%) [27, 28]. Most importantly, terbium-161 co-emits a substantial number of short-ranged electrons (conversion and Auger electrons) [25, 29], thought to be effective for the treatment of single cancer cells due to their high LET (4–26 keV/μm) [30–32]. Several preclinical studies using folate conjugates and ligands targeting the prostate-specific membrane antigen (PSMA) demonstrated the superior therapeutic effect of terbium-161 over lutetium-177 and the absence of additional side effects [33–35].

A central question in the context of using terbium-161 refers to the type of targeting agent which would ensure the greatest benefit from the effect of the co-emitted conversion and Auger electrons. It is commonly believed that nuclear localization of a peptide would be essential to induce DNA double-strand breaks (DSBs) by short-ranged electrons [36, 37]. It was, however, also shown that the cell membrane may be a suitable target for Auger electron emitters [38]. We believe that the non-internalizing SSTR antagonists may, thus, benefit from the co-emitted Auger electrons of terbium-161, which could eventually lead to a paradigm shift in PRRT.

The aim of this study was, therefore, to investigate the impact of the localization of ^161^Tb-based SST analogues to benefit from the short-ranged electrons in PRRT of NENs. The therapeutic efficacy of three ^161^Tb-labeled SST analogues supposed to localize (i) in the cytoplasm (DOTATOC, SSTR agonist [39]), (ii) in the cytoplasm and cell nucleus (DOTATOC-NLS [40, 41]) or at the cell membrane (DOTA-LM3, SSTR antagonist [23]) was compared with those of their ^177^Lu-labeled counterparts.

## Materials and methods

### Radiopeptide preparation

No-carrier-added (n.c.a.) terbium-161 was produced at Paul Scherrer Institute, Switzerland, as previously reported [42] and n.c.a. lutetium-177 was obtained from ITM Medical Isotopes GmbH, Germany. Radiolabeling and quality control of the SST analogues were performed as previously reported [43]. [^161^Tb]Tb-DOTATOC and [^177^Lu]Lu-DOTATOC, [^161^Tb]Tb-DOTATOC-NLS, and [^177^Lu]Lu-DOTATOC-NLS as well as [^161^Tb]Tb-DOTA-LM3 and [^177^Lu]Lu-DOTA-LM3 were obtained at radiochemical purity of ≥ 98% (up to 100 MBq/nmol) (Supplementary Material, Fig. S1/S2).

### Study design

Initially, the tumor uptake, internalization, and subcellular localization of the three SST analogues were assessed in SSTR-positive cancer cells. Afterwards, the therapeutic effect of the ^177^Lu- and ^161^Tb-labeled somatostatin analogues was investigated in vitro using cell viability and survival assays. In vivo, the biodistribution of the radiopeptides was determined under variable conditions, after which the most promising setting was used for an in vivo comparison of the therapeutic effects of the ^177^Lu- and ^161^Tb-labeled SST analogues.

Since it was previously confirmed that the SST analogues have equal in vitro and in vivo behavior regarding (sub)cellular uptake and tissue distribution, irrespective of whether they were labeled with lutetium-177 or terbium-161 [43], those experiments were performed with only one version of the radiolabeled peptides.

### AR42J tumor cell uptake and internalization

AR42J tumor cells, a SSTR-positive exocrine rat pancreatic cancer cell line [44] (ECACC 93,100,618, Health Protection Agency Culture Collections, Salisbury, UK), were cultured as previously reported (Supplementary Material) [43]. Cell uptake and internalization studies were performed to determine the fractions of radiopeptides localized at the membrane or in the cytoplasm, respectively. AR42J tumor cells were grown as monolayers in 12-well plates overnight, followed by incubation of the cells with each of the ^177^Lu-labeled radiopeptides (~ 15 kBq, ~ 0.375 pmol per well) for 2 h. The total uptake and internalization were determined after rinsing the cells with phosphate-buffered saline (PBS) and acidic glycine buffer, respectively. The uptake after SSTR saturation was evaluated after incubating the cells with increasing molar amounts of the SST analogues (Supplementary Material).

### Determination of the cellular localization of the radiopeptides

The nuclear accumulation of activity was determined after a 2 h-incubation period of the AR42J tumor cells with the respective radiopeptide using a nuclei isolation kit (Nuclei EZ Prep Kit, Sigma-Aldrich, St. Louis, U.S.) (Supplementary Material). The nuclei isolation assay was performed in triplicate with each ^177^Lu-labeled radiopeptide. The nuclear fraction of the respective radiopeptide was expressed as percentage of total cell uptake determined as described above.

### Cell viability and survival after treatment

The viability of AR42J tumor cells after incubation with ^161^Tb- or ^177^Lu-labeled DOTATOC, DOTATOC-NLS or DOTA-LM3 (0.001–40 MBq/mL, 0.01–400 pmol/mL) was determined using a 3-(4,5-dimethylthiazol-2-yl)-2,5-diphenyltetrazolium bromide (MTT) assay [33, 45]. The cell viability of treated cells was presented as average ± standard deviation (SD) of 4–5 independent experiments and expressed as percentage of sham-treated cells (set as 100%). The data were plotted against the applied activity concentration in logarithmic scale and fitted with a dose–response curve to determine the activity concentration necessary to reduce AR42J tumor cell viability to 50% of untreated control cells (EC_50_). The survival of the treated AR42J tumor cells was investigated using a clonogenic assay [46]. AR42J tumor cells were seeded as single cells in 6-well plates (2000 cells per well) and exposed to variable activity concentrations (0.01–5 MBq/mL, 0.33–170 pmol/mL) of ^161^Tb- and ^177^Lu-labeled SST analogues. Two hours later, the activity was removed and the cells were rinsed and let to grow into colonies over 2 weeks. The colonies were colored with crystal violet and counted as previously described (Supplementary Material) [46]. The survival of treated cells was expressed as average ± SD of 3 independent experiments performed in triplicates and expressed as percentage of sham-treated cells (set as 100%). The survival data were analyzed with a two-way ANOVA with Sidak’s multiple comparisons post-test.

### DNA damage evaluation

The number of DNA DSBs induced in AR42J tumor cells after exposure to the radiopeptides was assessed by immunostaining of γH2AX. The cells were treated in Petri dishes for 2 h using 2.5 MBq/mL or 10 MBq/mL of each radiopeptide followed by incubation with fresh culture medium for additional 24 h. Cell pellets obtained after centrifugation were fixed and embedded in paraffin and cut into sections. The immunostaining was performed using a phospho-histone H2A.X (Ser139) rabbit monoclonal antibody (Cell Signaling Techonology, Danvers, Massachusetts, USA) and an anti-rabbit, horseradish peroxidase-derivatized secondary antibody with DAB substrate buffer (Agilent Technologies, Santa Clara, California, USA) (Supplementary Material). The sections were scanned using a digital slide scanner (NanoZoomer-XR C12000; Hamamatsu, Japan) and the positively and negatively stained AR42J tumor cells were quantified with the pathology image analysis software VIS (Visiopharm Integrator System, Version 208 2019.02.2.6239, Visiopharm, Hoersholm, Denmark) (Supplementary Material). The percentage of γH2AX-positive cells in sham-treated samples was in average 1%. Data were analyzed with a one-way ANOVA with Dunnet’s multiple comparisons post-test.

### In vivo studies

The animal experiments were carried out according to the guidelines of Swiss Regulations for Animal Welfare, ethically approved by the Cantonal Committee of Animal Experimentation and permitted by the responsible cantonal authorities (license N° 75721 and 79692). Five-week-old female, athymic nude mice (CD-1 Foxn-1/nu) were obtained from Charles River Laboratories (Sulzfeld, Germany). Mice were subcutaneously inoculated with AR42J tumor cells (5 × 10^6^ cells in 100 µL PBS) for SPECT/CT imaging, biodistribution, and therapy studies.

### SPECT/CT imaging and biodistribution studies

The studies were performed 10–14 days after tumor cell inoculation when the tumor size reached a volume of ~ 250 mm^3^. [^161^Tb]Tb-DOTATOC, [^161^Tb]Tb-DOTATOC-NLS, or [^161^Tb]Tb-DOTA-LM3 (15 MBq, 1 nmol) was injected to acquire scans under isoflurane/oxygen anesthesia using a dedicated small-animal SPECT/CT scanner (NanoSPECT/CT, Mediso Medical Imaging Systems, Budapest, Hungary) as previously reported (Supplementary Material) [43]. In a first series of biodistribution studies, it was subsequently assessed that a molar peptide amount of 0.2 nmol per mouse was the optimum to achieve high tumor-to-background ratios (Supplementary Material). The time-dependent biodistribution studies were, therefore, performed with mice (*n* = 3 per group) intravenously injected with 0.2 nmol of the radiolabeled DOTATOC or DOTA-LM3 (5 MBq, in 100 μL PBS containing 0.05% bovine serum albumin (BSA)), respectively. Selected tissues and organs were collected, weighed, and counted for activity using a γ-counter (Perkin Elmer). The decay-corrected data were listed as a percentage of the injected activity per gram of tissue mass (% IA/g). The data were analyzed for significance using a two-way ANOVA with Tukey’s multiple comparisons post-test.

### Therapy study

The therapy study was initiated with mice randomly assigned to five groups (*n* = 6) when the AR42J tumors reached an average volume of 99 ± 16 mm^3^. At day 0 and day 7 of the study, the mice were intravenously injected with vehicle only (Group A: PBS with 0.05% BSA; sham-treatment), [^161^Tb]Tb-DOTATOC (Group B: 10 MBq, 0.2 nmol), [^177^Lu]Lu-DOTATOC (Group C: 10 MBq, 0.2 nmol), [^161^Tb]Tb-DOTA-LM3 (Group D: 10 MBq, 0.2 nmol), and [^177^Lu]Lu-DOTA-LM3 (Group E: 10 MBq, 0.2 nmol) (Table 1). The relative body weight (RBW) and the relative tumor volume (RTV) were defined based on the values at therapy start as previously described (Supplementary Material) [47]. The endpoint criteria were defined according to a scoring system which required euthanasia of mice with a score ≥ 3 (Supplementary Material).

**Table 1:** Design of the therapy study including the average tumor volumes and body weights of mice at therapy start. The mice were injected at day 0 and day 7 with the radiopeptide at 0.2 nmol per mouse (*n* = 6)

Group	Treatment	Injected activity	Tumor volume^b^	Body weight^c^
		day 0 and day 7	(mm^3^)	(g)
			(average ± SD)	(average ± SD)
		0.2 nmol/mouse	Day 0	Day 0
A	Vehicle^a^	-	118 ± 90	23 ± 2
B	[^161^Tb]Tb-DOTATOC	2 × 10 MBq	92 ± 48	24 ± 2
C	[^177^Lu]Lu-DOTATOC	2 × 10 MBq	102 ± 56	24 ± 2
D	[^161^Tb]Tb-DOTA-LM3	2 × 10 MBq	76 ± 32	23 ± 1
E	[^177^Lu]Lu-DOTA-LM3	2 × 10 MBq	109 ± 72	24 ± 2

^a^Vehicle: 0.05% BSA in PBS; ^b^no significant differences determined between the tumor volumes measured for each group (*p* > 0.05); ^c^no significant differences determined between the body weights measured for each group (*p* > 0.05)

### Assessment of the therapeutic efficacy

The efficacy of the treatment was assessed by comparison of the RTVs, measured every second day, of mice in each group using a two-way ANOVA with Sidak’s multiple comparisons post-test The average tumor growth delay, herein defined as the time during which the tumors did not grow or even decreased in size, was determined for mice of each group. For the subsequent phase, in which the tumors started to regrow, the doubling time of the tumor volume was calculated based on the fitted exponential tumor growth curve. The average ± SD of tumor growth delay and of the doubling time of the tumor volume in single mice, respectively, were compared among groups with a one-way ANOVA with Tukey’s multiple comparisons post-test. The survival times of mice were presented by Kaplan–Meier curves and analyzed using a log-rank test (Mantel-Cox).

### Assessment of early side effects

Early side effects were assessed based on the RBW of the mice. When an endpoint was reached or at the end of the study (at day 49), blood plasma parameters (albumin (ALB), creatinine (CRE), blood urea nitrogen (BUN), alkaline phosphatase (ALP), and total bilirubin (TBIL)) were determined (Supplementary Material). At the same time, relevant organs and tissues were collected, and the masses were put into relation to the brain mass of the respective mouse in order to allow the comparison of organ-to-brain and organ-to-body weight ratios among the single treatment groups (Supplementary Material). Data were analyzed for significance using a one-way ANOVA test with a Tukey’s multiple comparisons post-test.

### Statistical analysis and graphs preparation

The GraphPad Prism software (version 8) was used for preparation of the graphs, for the analysis of the data and to perform statistical analysis. A *p* value < 0.05 was considered statistically significant. SPECT/CT images were prepared using the software CorelDRAW (version X7).

## Results

### Tumor cell uptake and localization

**Fig. 1:**
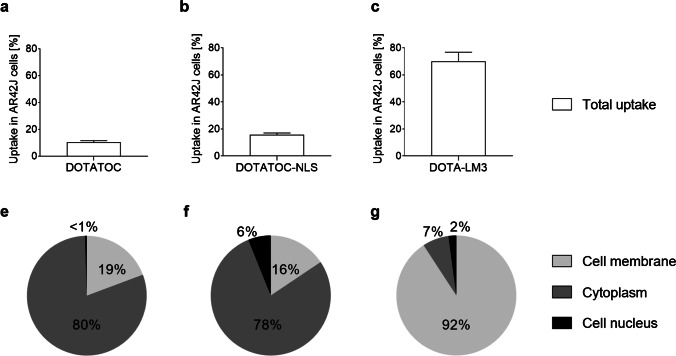
Graphs representing cell uptake and localization of **a/e** radiolabeled DOTATOC; **b/f** radiolabeled DOTATOC-NLS, and **c/g** radiolabeled DOTA-LM3

DOTA-LM3, irrespective of whether it was labeled with terbium-161 or lutetium-177 [43], showed the highest AR42J tumor cell uptake in vitro with ~70% of total added activity after a 2-h incubation period. This was 4- to sixfold higher than the uptake of radiolabeled DOTATOC (~ 10%) and DOTATOC-NLS (~ 15%), respectively (Fig. 1a–c); (Supplementary Material, Fig. S3).

About 9% of the cellular uptake of radiolabeled DOTA-LM3 was internalized whereas in the case of DOTATOC and DOTATOC-NLS, the internalized fraction was much higher (~ 81% and ~ 84%, respectively) (Fig. 1e–g). The fraction of accumulated radiopeptide in the cellular nucleus was ~ 6% of the total uptake in the case of radiolabeled DOTATOC-NLS but < 2% for radiolabeled DOTATOC and DOTA-LM3 (Fig. 1e–g).

### In vitro tumor cell viability

**Fig. 2:**
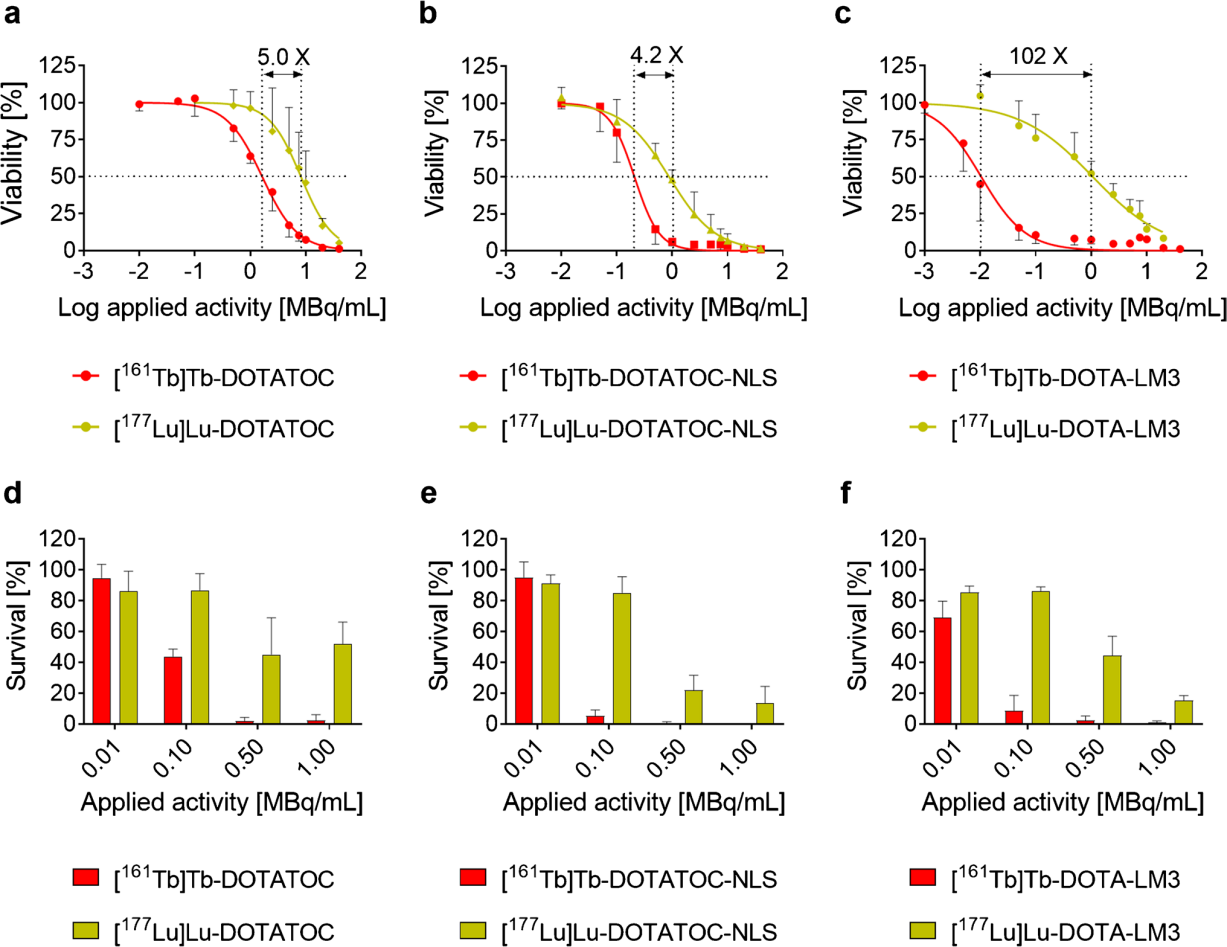
Graphs representing AR42J tumor cell viability and survival after treatment. **a/b/c** Results of the cell viability assessment (MTT assay); **d/e/f** results of the cell survival assessment (clonogenic assay)

It was observed that, in all cases, the ^161^Tb-labeled SST analogue was more potent in reducing AR42J tumor cell viability than the ^177^Lu-labeled analogue (Fig. 2a–c, Table S1, Supplementary Material). [^161^Tb]Tb-DOTATOC and [^161^Tb]Tb-DOTATOC-NLS were ~ 5- and ~ fourfold more potent than [^177^Lu]Lu-DOTATOC and [^177^Lu]Lu-DOTATOC-NLS, respectively, but [^161^Tb]Tb-DOTA-LM3 was 102-fold more potent than [^177^Lu]Lu-DOTA-LM3.

Importantly, [^161^Tb]Tb-DOTA-LM3, the most potent radiopeptide, revealed an EC_50_ of 0.010 MBq/mL (CI 0.008–0.014), which was 820-fold more potent than the clinically used [^177^Lu]Lu-DOTATOC (EC_50_ of 8.2 MBq/mL, CI 6.4–10).

### In vitro tumor cell survival

Colony-forming assays confirmed that the ^161^Tb-labeled peptides reduced AR42J tumor cell survival more effectively than the respective ^177^Lu-labeled analogues (Fig. 2d/e/f). Compared to untreated control cells, less than 3% of cells treated with 0.50 MBq/mL [^161^Tb]Tb-DOTATOC survived. To reach a similar effect with [^177^Lu]Lu-DOTATOC, a tenfold higher activity concentration had to be applied (data not shown). [^161^Tb]Tb-DOTATOC-NLS and [^161^Tb]Tb-DOTA-LM3 reduced the cell survival to < 5% at an activity concentration of 0.1 MBq/mL, while [^177^Lu]Lu-DOTATOC-NLS and [^177^Lu]Lu-DOTA-LM3 applied at a tenfold higher concentration reduced the survival to only ~ 13% and ~ 15%, respectively.

### DNA damage determined by the number of induced γH2AX foci

**Fig. 3:**
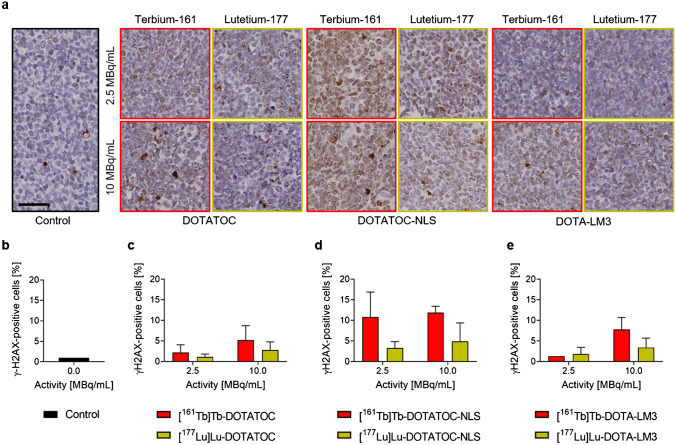
DSBs quantification (γH2AX) in AR42J cells. **a** Representative γH2AX-staining in sham-treated cells or cells treated with the respective radiopeptide (scale bar: 50 μm). Quantification of γH2AX staining in **b** sham-treated cells; or cells treated with **c** [^161^Tb]Tb-/[^177^Lu]Lu-DOTATOC; **d** [^161^Tb]Tb-/[^177^Lu]Lu-DOTATOC-NLS or **e** [^161^Tb]Tb-/[^177^Lu]Lu-DOTA-LM3. The number of positive cells is expressed in percent relative to the 1% positive cases detected in average in sham-treated cells

[^161^Tb]Tb-DOTATOC showed a tendency of inducing a higher number of DNA DSBs compared to [^177^Lu]Lu-DOTATOC (*p* > 0.05) (Fig. 3). Exposure of tumor cells to [^161^Tb]Tb-DOTATOC-NLS increased the number of γH2AX foci more than [^177^Lu]Lu-DOTATOC-NLS (> 11% γH2AX-positive cells vs ~ 5% at an activity concentration of 10 MBq/mL, *p* > 0.05). When applied at 2.5 MBq/mL, [^161^Tb]Tb-DOTA-LM3 had a similar effect as [^177^Lu]Lu-DOTA-LM3 (*p* > 0.05), but at 10 MBq/mL, it induced more DNA DSBs than [^177^Lu]Lu-DOTA-LM3 (~ 8% γH2AX-positive cells vs ~ 3%, *p* > 0.05).

### SPECT/CT imaging studies

**Fig. 4:**
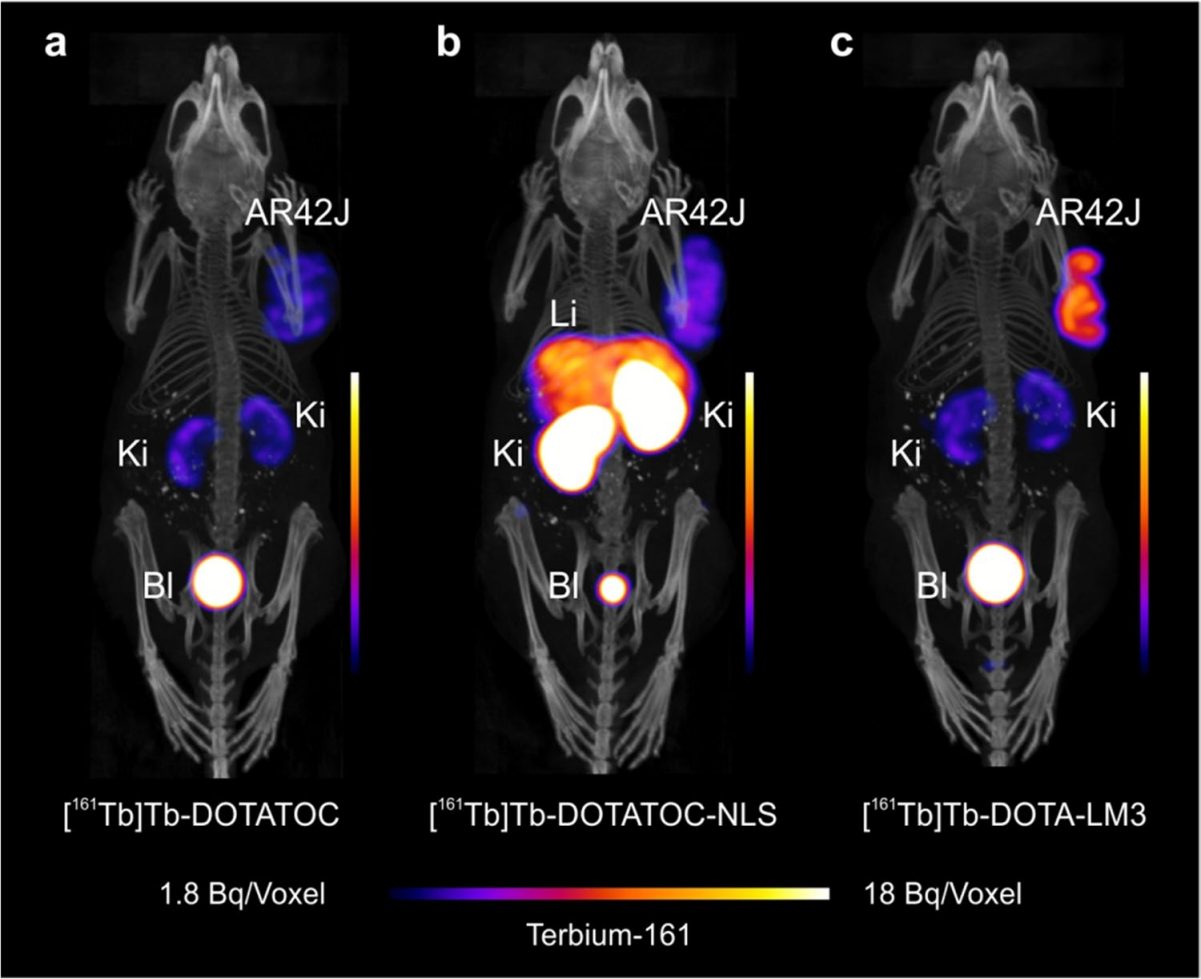
SPECT/CT images of AR42J tumor-bearing mice shown as maximum intensity projections 2 h after injection of the radiopeptides (15 MBq, 1.0 nmol per mouse). Mice injected with **a** [^161^Tb]Tb-DOTATOC [43]; **b** [^161^Tb]Tb-DOTATOC-NLS (b), and **c** [^161^Tb]Tb-DOTA-LM3 [43]. AR42J = SSTR-positive xenograft; Ki = kidneys; Li = liver; Bl = urinary bladder

As previously reported [43], [^161^Tb]Tb-DOTATOC accumulated in the tumors, but retention in the kidneys was also visible on the 2 h p.i.-SPECT/CT scan (Fig. 4a). At the same timepoint after injection of [^161^Tb]Tb-DOTATOC-NLS, the tumor uptake was slightly increased, but retention in the liver and kidneys was exceedingly high. As a result, this radiopeptide would not be applicable for therapeutic purposes (Fig. 4b) (Supplementary Material, Fig. S4). [^161^Tb]Tb-DOTA-LM3 showed the most favorable tissue distribution, with an increased tumor uptake, resulting in the highest tumor-to-kidney ratios (Fig. 4c) [43]. The tumor uptake was SSTR-specific for all radiopeptides, as previously reported (Supplementary Material) [43].

### Biodistribution studies

**Fig. 5:**
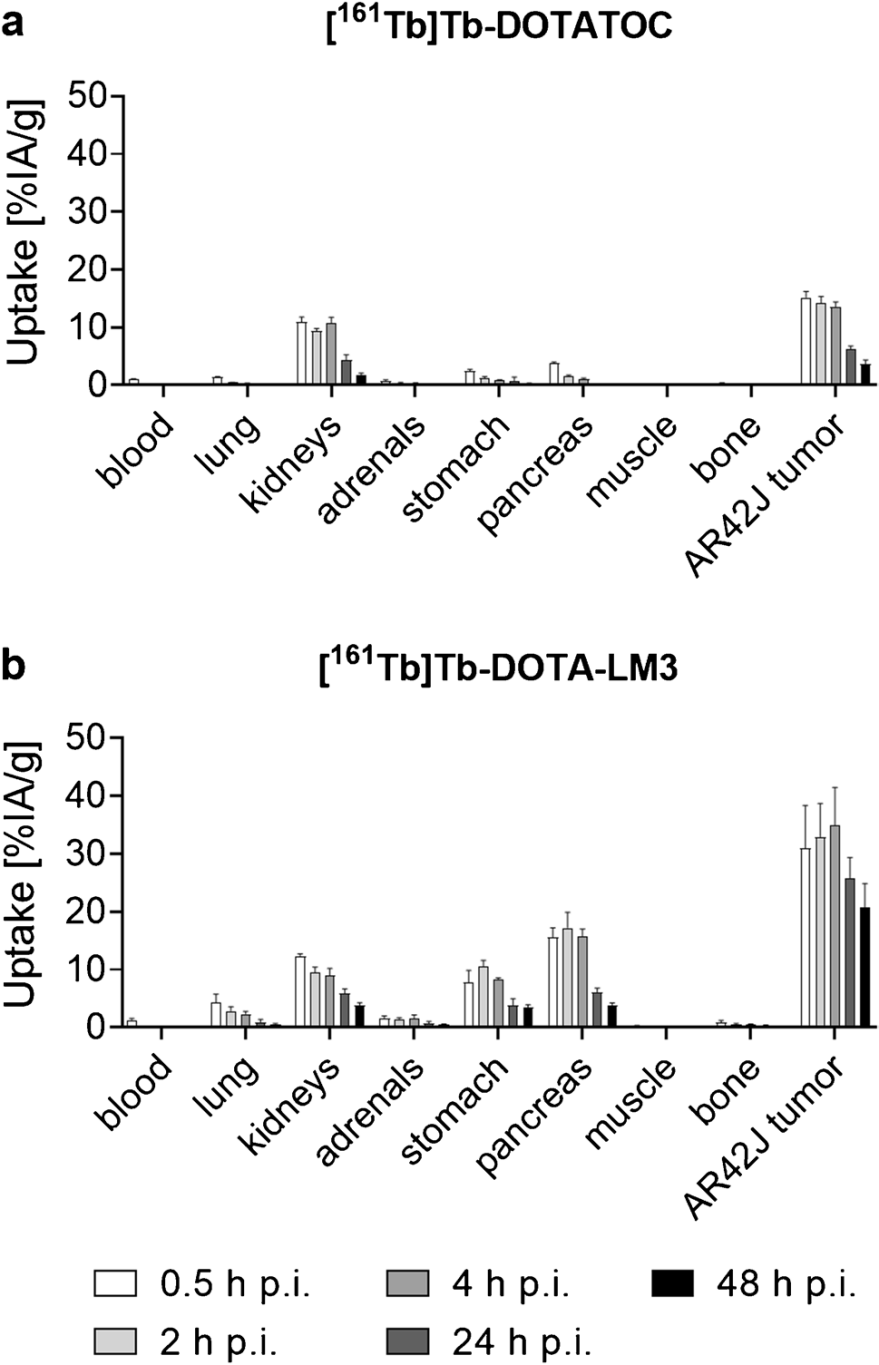
Biodistribution data obtained in AR42J tumor-bearing mice. Results after injection of **a** [^161^Tb]Tb-DOTATOC (0.2 nmol/mouse) and **b** [^161^Tb]Tb-DOTA-LM3 (0.2 nmol/mouse) shown in percentage of injected activity per tissue mass (% IA/g) and presented as the average ± SD of *n* = 3

Time-dependent biodistribution studies were performed with radiolabeled DOTATOC and DOTA-LM3 using the peptide amount (0.2 nmol per mouse) that was evaluated in this work as most favorable to achieve high tumor-to-background ratios (Supplementary Material, Fig. S5, Tables S2/S3). Due to the unfavorable in vivo distribution of [^161^Tb]Tb-DOTATOC-NLS with high uptake in liver and kidneys, as demonstrated by SPECT/CT imaging, DOTATOC-NLS was excluded from further in vivo studies. [^161^Tb]Tb-DOTATOC reached the highest tumor uptake early after injection (15 ± 1% IA/g; 0.5 h p.i.), which was retained over the following 4 h, but dropped afterward to 6.3 ± 0.6% IA/g (24 h p.i.) and 3.7 ± 0.7% IA/g (48 h p.i.). Significant activity accumulation was also observed in the lungs, stomach, and pancreas shortly after injection; however, it was effectively cleared over time (≤ 1%IA/g; 4 h p.i.). Renal clearance was slower (~ 10% IA/g; 4 h p.i. and ~ 4–5% IA/g at 24 h p.i.) (Fig. 5a; Supplementray Material Table S4).

The tumor uptake and retention of [^161^Tb]Tb-DOTA-LM3 was significantly higher (35 ± 7% IA/g; 4 h p.i.; 21 ± 4%IA/g at 48 h p.i.) than for [^161^Tb]Tb-DOTATOC at all investigated timepoints (*p* < 0.05). In the lungs, stomach, and pancreas, activity was detected during the first 4 h but effectively cleared over the hours that followed. The accumulated activity of [^161^Tb]Tb-DOTA-LM3 in the pancreas and stomach was significantly higher than in the case of [^161^Tb]Tb-DOTATOC (*p* < 0.05) at all investigated timepoints, whereas renal uptake was similar as observed for [^161^Tb]Tb-DOTATOC (*p* > 0.05) (Fig. 5b and Supplementary Material Table S5). As previously demonstrated [43], the in vivo distribution of ^161^Tb- and ^177^Lu-labeled SST-analogues is equal; hence, the results hold true irrespective of which radionuclide was employed.

### Therapy study in AR42J tumor-bearing mice

**Fig. 6:**
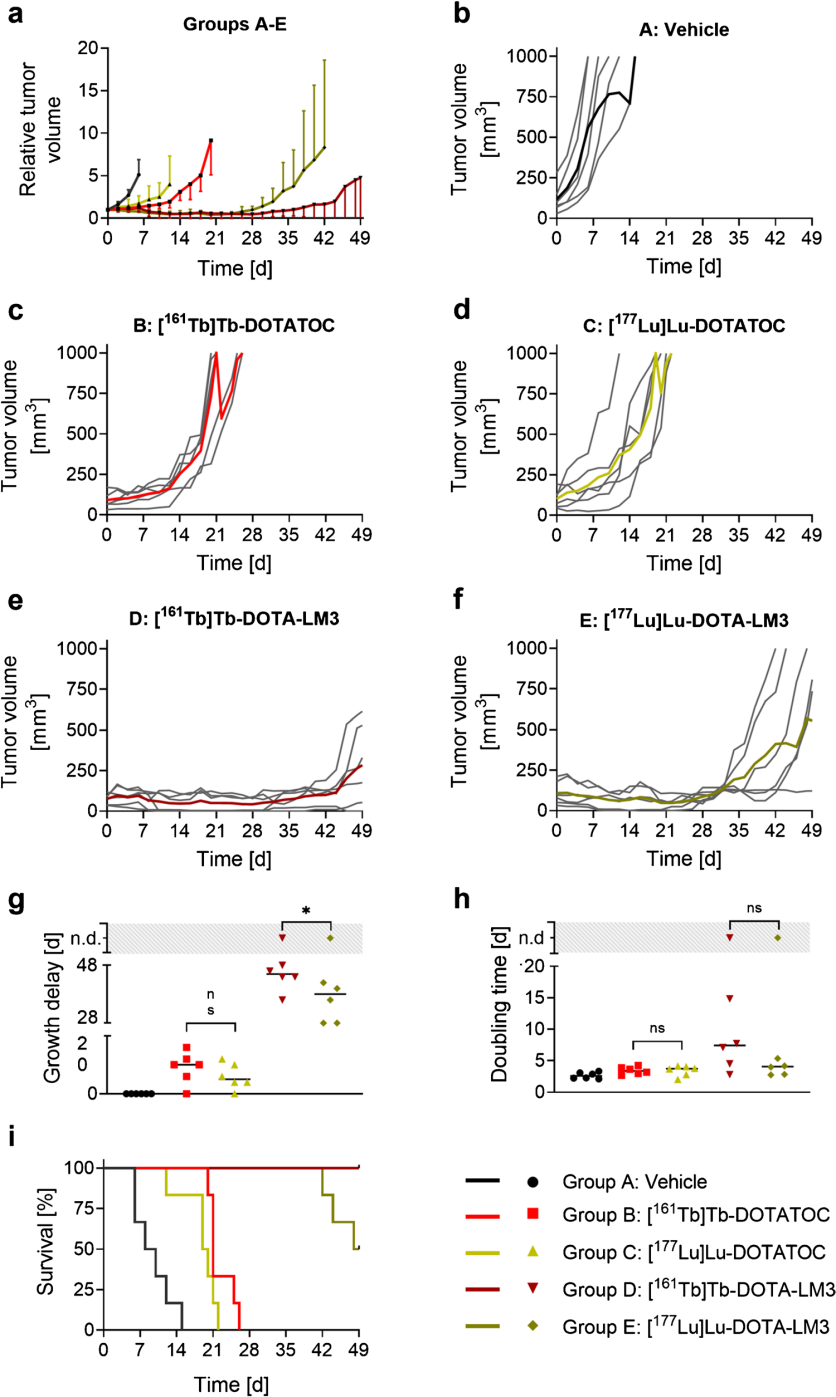
Results of the therapy study performed with ^161^Tb- and ^177^Lu-SSTR agonist and antagonist (2 × 10 MBq; 0.2 nmol) in AR42J tumor-bearing mice. **a** The RTV at day 0 (set as 1) for mice of Group A (sham), B ([^161^Tb]Tb-DOTATOC), C ([^177^Lu]Lu-DOTATOC), D ([^161^Tb]Tb-DOTA-LM3), and E ([^177^Lu]Lu-DOTA-LM3). Data are shown until the first mouse of the respective group reached an endpoint. **b**/**c**/**d**/**e**/**f** Absolute TV of single mice (grey lines) and average (colored line) of Groups A–E; **g** tumor growth delay of Group A–E; **h** tumor doubling time of Groups A–E; **i** Kaplan–Meier plot of Groups A–E

Sham-treated mice of Group A showed an exponential tumor growth so that the endpoint was reached within the first 14 days in all cases. The tumor growth was delayed in treated mice of Groups B–E, resulting in significantly prolonged median survival times compared to the 9 days in the control group (Fig. 6, Table 2). Mice treated with [^161^Tb]Tb-DOTATOC (Group B) showed a slightly slower tumor growth compared to mice treated with [^177^Lu]Lu-DOTATOC (Group C). After 12 days from the therapy start, the RTV of mice of these two groups were significantly different (2.0 ± 0.7 vs 4.0 ± 3.3, *p* < 0.05). The tumor growth delay and doubling time were higher in Group B (9.0 ± 5.5 days and 3.4 ± 3.6 days, respectively) compared to those of Group C (6.0 ± 4.4 days and 3.4 ± 0.8 days, respectively, *p* > 0.05). Mice of Group B were, thus, euthanized at a later stage (day 20–26) compared to Group C (day 12–22). The median survival (21 vs 19.5 days) was, however, comparable between the groups (*p* > 0.05).

**Table 2:** Data regarding euthanasia period and median survival

Group	Treatment	Time frame of euthanasia	Median survival
		[day]	[day]
A	Vehicle	6–15	9
B	[^161^Tb]Tb-DOTATOC^a^	20–26	21
C	[^177^Lu]Lu-DOTATOC^a^	12–22	19.5
D	[^161^Tb]Tb-DOTA-LM3^a^	end of study (*n* = 6)	> > 49^b^
E	[^177^Lu]Lu-DOTA-LM3^a^	42–48 (*n* = 3) end of study (*n* = 3)	48.5

^a^1^st^ injection: 10 MBq, 0.2 nmol at day 0; 2^nd^ injection: 10 MBq, 0.2 nmol at day 7.

^b^End of study at day 49.

The tumor growth delay for mice treated with [^161^Tb]Tb-DOTA-LM3 was 44 ± 5 days, but only 35 ± 7 days in mice treated with [^177^Lu]Lu-DOTA-LM3 (*p* < 0.05). Afterwards, the tumors started to regrow exponentially in 5 out of 6 mice of both groups; however, the doubling time was considerably longer for mice treated with [^161^Tb]Tb-DOTA-LM3 compared to the tumor growth in mice treated with [^177^Lu]Lu-DOTA-LM3 (7.4 ± 4.6 days vs 3.8 ± 1.1 days, *p* > 0.05). All mice treated with [^161^Tb]Tb-DOTA-LM3 survived until the end of the study, while this was the case only for three out of six mice treated with [^177^Lu]Lu-DOTA-LM3.

### Assessment of potential early side effects

**Fig. 7:**
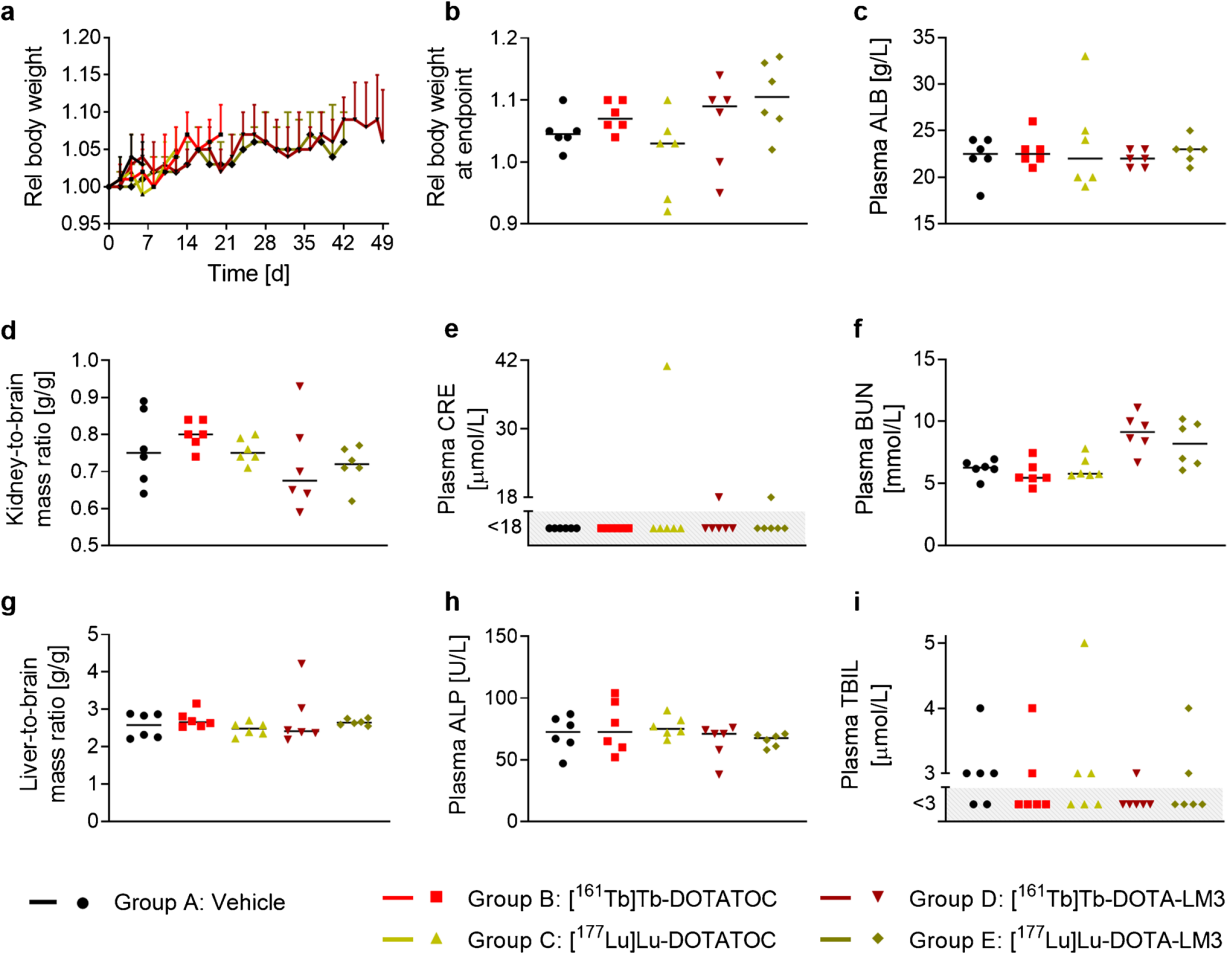
Parameters of potential side effects in therapy mice. **a**/**b**/**c** Indicators for the general health status of the mice: RBW during the therapy (**a**) shown until the first mouse of the group reached an endpoint, RBW at the endpoint (**b**), and plasma ALB levels (**c**). **d**/**e**/**f** Indicators for kidney toxicity: kidney-to-brain mass ratios (**d**), plasma CRE (**e**), plasma BUN levels (**f**). **g**/**h**/**i** Indicators for liver toxicity: liver-to-brain mass ratios (**g**), plasma ALP levels (**h**), and plasma TBIL levels (**i**). Organ-to-brain mass ratios and plasma parameters refer to mice at the endpoint

No signs of early side effects were detected in any of the treated groups (Fig. 7, Supplementary Material, Tables S6–S9). The mice gained weight over the course of the study and the body weights did not differ between the groups at any timepoint (*p* > 0.05) (Fig. 7a/b). ALB plasma levels, an indicator of general health status of mice, were comparable among the groups (Fig. 7c). No signs of kidney toxicity were observed at the time of euthanasia, as kidney-to-brain mass ratios and plasma CRE levels showed no significant difference among the groups with the exception of one outlier (Fig. 7d/e). The BUN levels were elevated in mice treated with [^161^Tb]Tb-DOTA-LM3 (9.9 ± 1.5 mmol/L) and [^177^Lu]Lu-DOTA-LM3 (8.2 ± 1.9 mmol/L) compared to control mice (6.2 ± 0.7 mmol/L) and mice treated with [^161^Tb]Tb- or [^177^Lu]Lu-DOTATOC, but in the physiological range reported for this particular mouse strain by the breeding company (Charles River, Germany) (Fig. 7f). No liver toxicity was observed based on liver-to-brain mass ratios, plasma ALP, and TBIL which were comparable among all groups (*p* > 0.05) (Fig. 7g/h/i).

## Discussion

Terbium-161 and lutetium-177 share similar physical decay properties in terms of half-life and β¯-energy; however, the co-emission of short-ranged electrons is a specific characteristic of terbium-161. A direct comparison of the therapeutic effect of terbium-161 and lutetium-177 is feasible due to the similar chemical properties of these radiolanthanides and, thus, equal tumor cell uptake and biodistribution profiles of the investigated pairs of radiopeptides herein [43]. In this study, we investigated the impact of the subcellular localization of SST analogues on the therapeutic efficacy of terbium-161 and lutetium-177, respectively. Both DOTATOC and DOTATOC-NLS internalized to a large degree into the cytoplasm (80% and 84%) whereof, in the case of DOTATOC-NLS, ~ 15% of the internalized fraction were shuttled to the nucleus. In contrast, the largest fraction of the SSTR antagonist DOTA-LM3 remained at the cell membrane (> 90%).

Indeed, terbium-161 was more effective in reducing AR42J tumor cell viability and survival than lutetium-177, irrespective of the SST analogue employed. This indicated clearly that the conversion and Auger electrons emitted by terbium-161 contributed positively to its therapeutic efficacy. The difference in therapeutic efficacy between ^161^Tb- and ^177^Lu-labeled peptides was, however, not the same among the three types of SST analogues.

In line with the common belief that Auger electron emitters should be shuttled in close proximity to the cell DNA [36, 37], [^161^Tb]Tb-DOTATOC-NLS, which accumulated to about 6% in the cell nucleus, induced the highest number of DNA DSBs after 24 h incubation. [^161^Tb]Tb-DOTATOC and [^161^Tb]Tb-DOTA-LM3 induced the formation of far fewer DSBs at this timepoint. Despite the positive effect of the NLS to induce DSBs, the overall advantage of using [^161^Tb]Tb-DOTATOC-NLS instead of [^177^Lu]Lu-DOTATOC-NLS was similar to the advantage of [^161^Tb]Tb-DOTATOC over [^177^Lu]Lu-DOTATOC in both viability and survival assays. These findings, together with the unfavorably high uptake of [^161^Tb]Tb/[^177^Lu]Lu-DOTATOC-NLS in kidneys and liver, clearly indicated that the functionalization of a SST analogue with a NLS is not a feasible strategy to ideally exploit the therapeutic potential of terbium-161.

Interestingly, the non-internalizing [^161^Tb]Tb-DOTA-LM3 revealed a 102-fold increased efficacy to reduce cell viability in vitro compared to [^177^Lu]Lu-DOTA-LM3 (Fig. 2). These findings were in line with a previous observation made with internalizing and non-internalizing antibodies, wherof the latter were more effective in killing tumor cells when labeled with the Auger electron-emitting iodine-125 [38, 48, 49]. Obviously, the cell membrane is a better target to exploit the effect of short-ranged electrons than the cytoplasm. Current endeavors in our laboratories focus, thus, on a further radiobiological investigation of the effect of [^161^Tb]Tb-DOTA-LM3 to better understand the “membrane effect” of short-ranged electrons.

Preclinical therapy studies with AR42J tumor-bearing mice confirmed the superior efficacy of ^161^Tb-labeled SST analogues over those labeled with lutetium-177. The obvious positive effect of Auger electrons emitted by terbium-161 was particularly pronounced when using DOTA-LM3. The overall efficacy of DOTA-LM3-based PRRT was much better than the results obtained with DOTATOC, irrespective of which radionuclide was applied. It can be ascribed to the higher tumor accumulation of SSTR antagonists compared to agonists. [^161^Tb]Tb-DOTA-LM3 induced a tumor growth delay of over 44 days and was, thus, the most powerful candidate, with a significant advantage over the clinically applied [^177^Lu]Lu-DOTATOC, which induced a tumor growth delay of only 6 ± 4 days. No significant early side effects nor signs of liver or kidney toxicity were observed in any of the treated mice, yet further investigations are necessary in order to assess potentially negative effects to SSTR-expressing normal tissues or bone marrow toxicity.

Even though AR42J tumor cells show neuroendocrine properties [50] and have, thus, been extensively employed for the evaluation of SST analogues, it can be considered a limitation of this study to have only used rat tumor cells. It will, thus, be crucial to confirm the reported findings using human endocrine pancreatic tumor cells. The investigation of further “agonist/antagonist” pairs that target SSTR or other receptors will provide clarification about the general validity of the proposed advantage of terbium-161 when applied in combination with non-internalizing tumor-targeting agents. Both topics are in the focus of our currently-ongoing research activities.

Our previous work already showed that terbium-161 provides an advantage over the use of lutetium-177 [33–35]. If the concept presented herein will be confirmed in follow-up studies, terbium-161 may be used preferentially with non-internalizing tumor-targeting agents, thereby, complementing the use of α-particle emitters, which should be combined with fast-internalizing molecules.

Finally, our results, together with the recently reported data of the successful application of [^177^Lu]Lu-DOTA-LM3 in patients [24, 51], encourage a clinical translation of [^161^Tb]Tb-DOTA-LM3 for the treatment of NEN’s. Current efforts at PSI are, therefore, dedicated to the scale-up of the terbium-161 production, which is performed in analogy to the production of n.c.a. lutetium-177 (Supplementary Material, Fig. S6) [25, 42] to enable the preparation of therapeutic quantities of [^161^Tb]Tb-DOTA-LM3 under good manufacturing practice (GMP) for clinical application.

## Conclusion

This study showed that the cellular localization of ^161^Tb-labeled SST analogues is relevant to exploit the therapeutic potential of short-ranged electrons. [^161^Tb]Tb-DOTA-LM3 revealed to be powerful for the treatment of NENs, as it combines the favorable properties of SSTR antagonists with the advantages of terbium-161. Should our results be confirmed with other tumor models and targeting concepts, these results may initiate a paradigm shift towards the application of Auger electron emitters with membrane-targeting biomolecules to enable more effective PRRT in NENs.

## Supplementary Information

Below is the link to the electronic supplementary material.

Supplementary file1 (DOCX 3.82 MB)

## Data Availability

The raw data presented in this study are available on request from the corresponding author.
