# Supplementary material for: Combination of terbium-161 with somatostatin receptor antagonists—a potential paradigm shift for the treatment of neuroendocrine neoplasms
Source: Eur J Nucl Med Mol Imaging. 2021 Oct 8;49(4):1113–26. doi: 10.1007/s00259-021-05564-0 (PMC8921065; doi:10.1007/s00259-021-05564-0)
Supplement: Supplementary file 1 — Supplementary file1 (DOCX 3.82 MB) [file 259_2021_5564_MOESM1_ESM.docx]

**SUPPLEMENTARY MATERIAL**

**Combination of terbium-161 with somatostatin receptor antagonists – A potential paradigm shift for the treatment of neuroendocrine neoplasms**

Francesca Borgna^1^, Stephanie Haller^1^, Josep M. Monné Rodriguez^2^, Mihaela Ginj^3^, Pascal V. Grundler^1^, Jan Rijn Zeevaart^4^, Ulli Köster^5^, Roger Schibli^1,6^, Nicholas P. van der Meulen^1,7^, Cristina Müller^1,6*^

1. Center for Radiopharmaceutical Sciences ETH-PSI-USZ, Paul Scherrer Institute, 5232 Villigen-PSI, Switzerland

2. Laboratory for Animal Model Pathology, Institute of Veterinary Pathology, Vetsuisse Faculty, University of Zurich, 8057 Zurich, Switzerland

3. The Joint Department of Medical Imaging, University Health Network, 200 Elizabeth St., Toronto, Ont Canada M5G 2C4

4. Radiochemistry, South African Nuclear Energy Corporation (Necsa), Pelindaba, Brits, South, 0240, Africa

5. Institut Laue-Langevin, 38042 Grenoble, France

6. Department of Chemistry and Applied Biosciences, ETH Zurich, 8093 Zurich, Switzerland

7. Laboratory of Radiochemistry, Paul Scherrer Institute, 5232 Villigen-PSI, Switzerland

***Correspondence to**:

PD Dr. Cristina Müller

Center for Radiopharmaceutical Sciences ETH-PSI-USZ

Paul Scherrer Institute

5232 Villigen-PSI

Switzerland

e-mail: [cristina.mueller@psi.ch](mailto:cristina.mueller@psi.ch)

phone: +41-56-310 44 54

fax: +41-56-310 28 49

**1. Radionuclides and peptides**

Terbium-161 was produced using the ^160^Gd(n,γ)^161^Gd→^161^Tb nuclear reaction as previously reported [1, 2]. The irradiation of the targets was carried out at the SAFARI-1 reactor at Necsa in Pelindaba, South Africa or at High Flux Reactor at Institut Laue-Langevin in Grenoble, France, or at the spallation-induced neutron source SINQ, Villigen-PSI, Switzerland. The chemical separation of terbium-161 was performed at PSI as previously reported [2]. Terbium-161 was made available as no-carrier-added (n.c.a.) [^161^Tb]TbCl_3_ in 0.05 M HCl. Lutetium-177 was obtained as n.c.a. [^177^Lu]LuCl_3_ in 0.04 M HCl from ITM Medical Isotopes GmbH, Germany.

Terbium-161 and lutetium-177 were used for the evaluation of the somatostatin (SST) analogues, DOTATOC, DOTATOC-NLS, and DOTA-LM3 (Fig. S1). DOTA-[Tyr^3^]-octreotide (DOTATOC) was provided by ITM GmbH, Germany. DOTATOC modified with a NLS sequence (DOTATOC-NLS), based on a PKKKRKV peptide, was previously synthesized by Dr. Mihaela Ginj [3, 4] and kindly provided by Prof. Helmut R. Mäcke (Department of Nuclear Medicine, University Hospital of Freiburg, Freiburg, Germany). DOTA-LM3 was obtained as a custom synthesis by CSBio (Silicon Valley Menlo Park, California, U.S.A.) based on the structure published by Fani et al. [5].

**
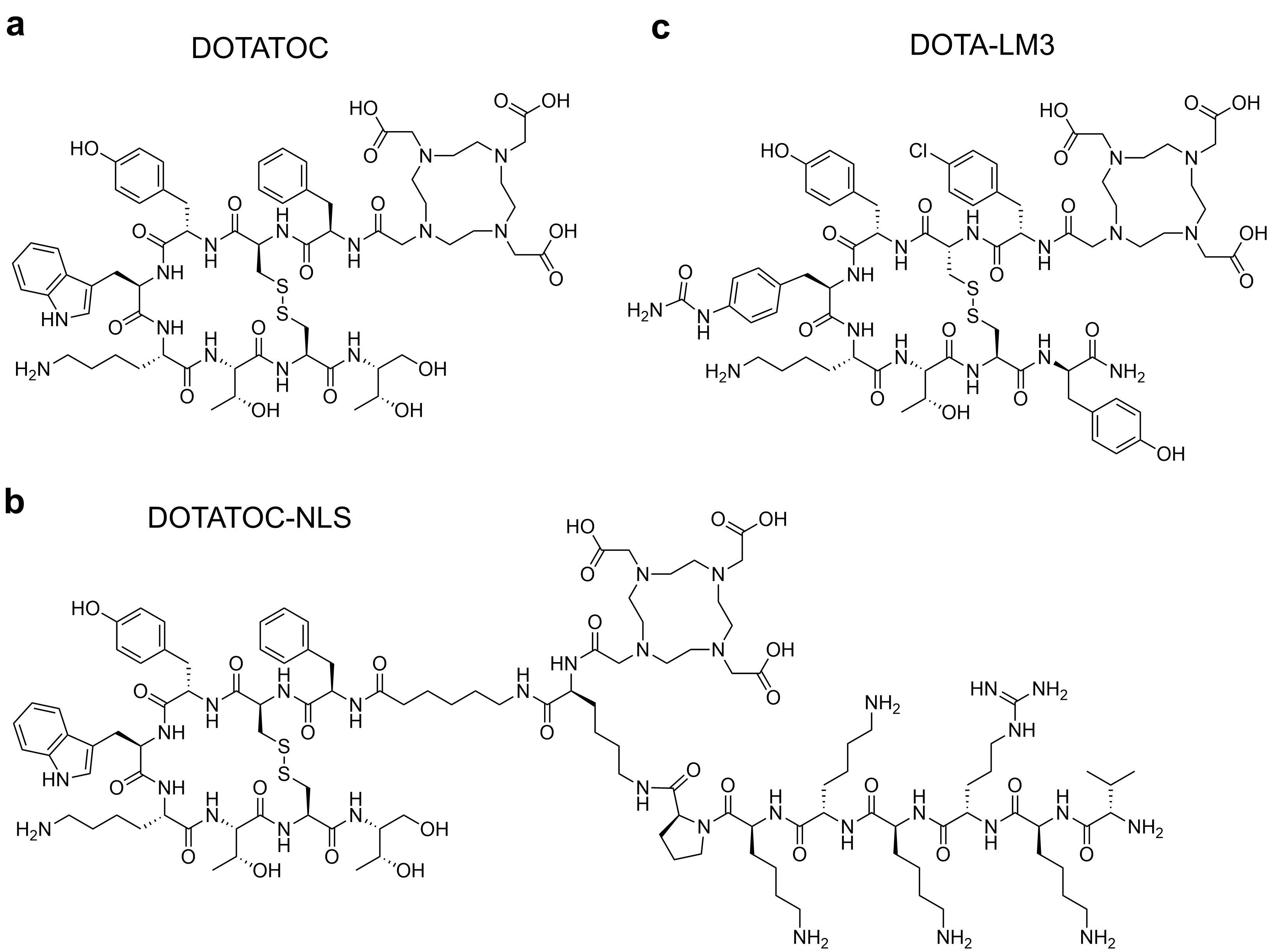
**

**Fig. S1** Chemical structures of the somatostatin (SST) analogues. (**A**) DOTATOC (somatostatin receptor (SSTR) agonist) [6, 7]; (**B**) DOTATOC-NLS (SSTR agonist equipped with a nuclear localization sequence) [3, 4]; (**C**) DOTA‐LM3, (SSTR antagonist) [5, 8]

**2. Preparation and in vitro evaluation of the radiopeptides**

***Purpose:*** The SST analogues were labeled with either terbium-161 or lutetium-177 to obtain [^161^Tb]Tb-DOTATOC and [^177^Lu]Lu-DOTATOC, [^161^Tb]Tb-DOTATOC-NLS and [^177^Lu]Lu-DOTATOC-NLS as well as [^161^Tb]Tb-DOTA-LM3 and [^177^Lu]Lu-DOTA-LM3.

***Methods:*** Stock solutions of DOTATOC**,** DOTATOC-NLS and DOTA-LM3, were prepared in Milli-Q water to obtain a final concentration of 1 mM. The SST analogues were labeled with terbium-161 or lutetium-177 at a molar activity up to 100 MBq/nmol using a 1:5 (*v/v*) mixture of sodium acetate (0.5 M) and HCl (0.05 M) at pH ~4.5 as previously reported [9-11]. The reaction mixture was incubated for 10 min at 95 °C, followed by a quality control using HPLC. For this purpose, a Merck Hitachi LaChrom HPLC system, equipped with a D-7000 interface, a L-7200 autosampler, a radioactivity detector (LB 506 B; Berthold) and a L-7100 pump connected with a reversed-phase C18 column (Xterra^TM^ MS, C18, 5 μm, 150 x 4.6 mm; Waters) was used. The mobile phase consisted of 0.1% (*v*/*v*) TFA in Milli-Q water (A) and acetonitrile (B). A linear gradient of solution A (95–20%) and solvent B (5–80%) over 15 min was used at a flow rate of 1.0 mL/min. The radiopeptides were diluted in Milli-Q water containing pentasodium diethylenetriaminepentaacetate (Na_5_-DTPA 50 µM) prior to injection into HPLC.

***Results:*** [^161^Tb]Tb-DOTATOC and [^177^Lu]Lu-DOTATOC, [^161^Tb]Tb-DOTATOC-NLS and [^177^Lu]Lu-DOTATOC-NLS as well as [^161^Tb]Tb-DOTA-LM3 and [^177^Lu]Lu-DOTA-LM3, prepared at a molar activity of up to 100 MBq/nmol, were obtained with radiochemical purity of ≥98% (Fig. S2).

***Remark:*** Since the uptake of radiolabeled peptides was identical, irrespective of whether they were labeled with terbium-161 or lutetium-177 [11], the in vitro and in vivo experiments not referring to the therapeutic effect were carried out using only one version of the radiolabeled peptides.


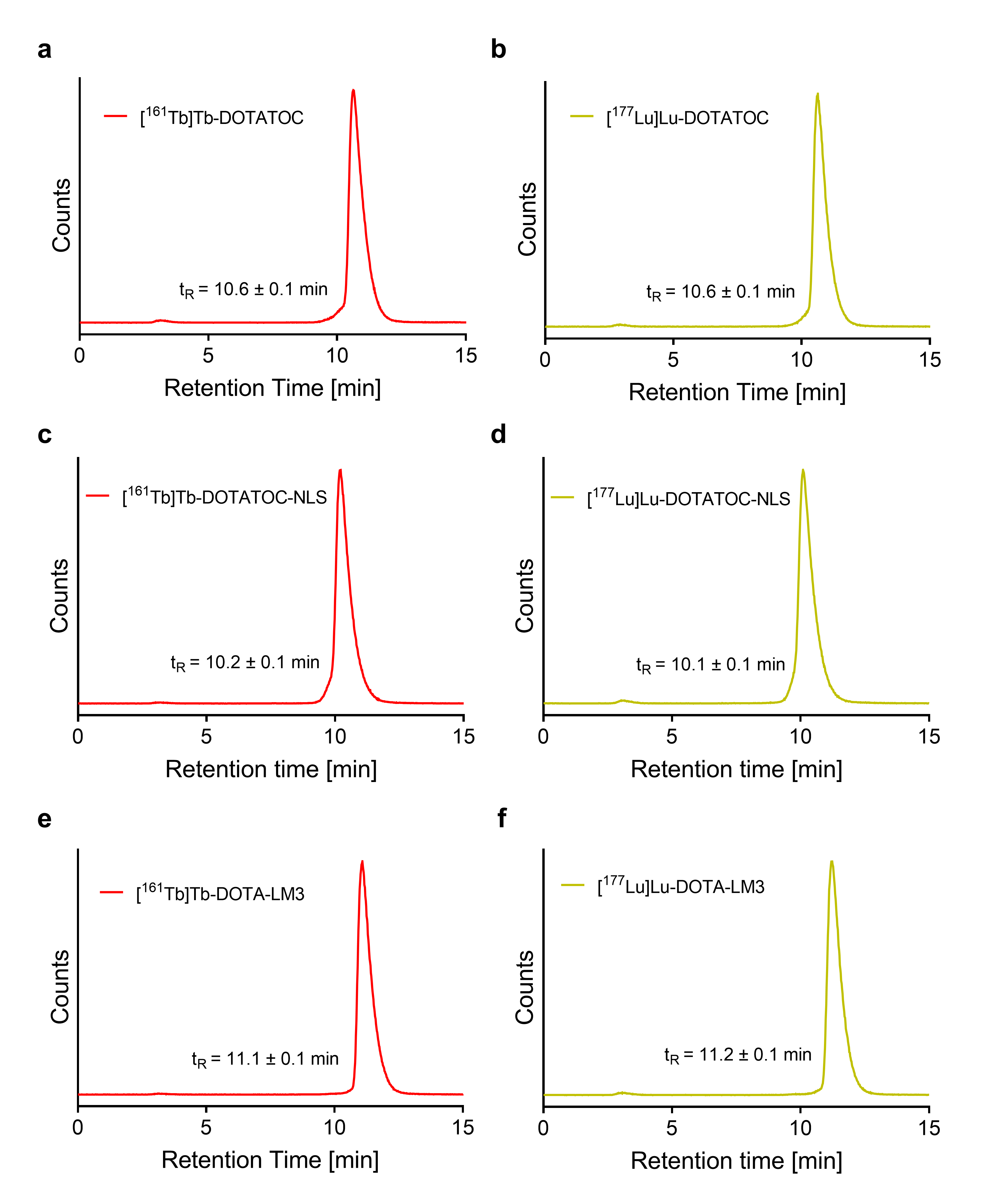


**Fig. S2** Representative HPLC chromatograms of the ^161^Tb- and ^177^Lu-labeled peptides. (**a**) [^161^Tb]Tb-DOTATOC^1)^; (**b**) [^177^Lu]Lu-DOTATOC^1)^; (**c**) [^161^Tb]Tb-DOTATOC-NLS; (**d**) [^177^Lu]Lu-DOTATOC-NLS; (**e**) [^161^Tb]Tb-DOTA-LM3^1)^; (**f**) [^177^Lu]Lu-DOTA-LM3^1)^. Traces of unreacted terbium-161 or lutetium-177 appeared with a retention time of ~2.5 min. ^1)^Reproduced with permission from Borgna et al., 2021 [11]

**3. Cell culture**

AR42J tumor cells, an SSTR-positive exocrine rat pancreatic cancer cell line, were cultured in RPMI 1640 culture medium supplemented with glutamine, antibiotics and 20% fetal calf serum (FCS), as previously reported [11]. Polystyrene well-plates were coated with poly-L-lysine (0.5 mg/mL) for all in vitro experiments to facilitate cell adhesion and prevent adherence of the radiopeptides to the well-plate material. Cell culture medium containing glutamine and antibiotics but only 1% FCS (referred herein as “assay medium”) was used for all in vitro assays. Incubation of cells always referred to standard culture conditions of a humidified atmosphere at 37 °C and 5% CO_2_ if not otherwise indicated.

**4. In vitro tumor cell uptake and internalization**

***Purpose:*** The goal of these in vitro studies was to determine the total cell uptake and internalization of the radiopeptides in AR42J tumor cells. In addition, the peptide amount, which resulted inSSTR saturation, was investigated using variable amounts of DOTATOC, DOTATOC-NLS and DOTA-LM3.

***Methods:*** Cell uptake and internalization studies were performed according to a previously published procedure [11]. Briefly, AR42J tumor cells (10^6^ cells/2 mL) were grown as a monolayer overnight at 37 °C and 5% CO_2_. After rinsing the cells with phosphate buffered saline (PBS) pH 7.4, they were incubated with [^177^Lu]Lu-DOTATOC, [^177^Lu]Lu-DOTATOC-NLS or [^177^Lu]Lu-DOTA-LM3 (25 μL, ~15 kBq 0.375-75 pmol). The radiopeptides were incubated for 2 h at 37 °C. To determine the total cell uptake, the supernatants were removed and the cells rinsed three times with ice-cold PBS. The internalized fraction was determined by stripping the cells with a glycine-based acidic buffer (pH 2.8) for 30 min followed by rinsing the cells with ice-cold PBS. Cell samples were lysed by addition of NaOH (1 M, 1 mL) to each well. The cell lysates were measured for activity using a γ-counter (Perkin Elmer, Wallac Wizard 1480). The activity of the samples was standardized to the average protein concentration in each well (~0.3 mg) using a Micro BCA Protein Assay kit (Pierce, Thermo Scientific). Experiments were performed twice in triplicate.

***Results:*** In all cases, the uptake and internalization of the respective radiopeptide decreased while increasing the molar amount of non-labeled peptide (Fig. S3). Saturation was not observed at 0.375 pmol and 0.75 pmol peptide amount, but the uptake and internalization of [^177^Lu]Lu-DOTATOC was decreased by ~30% when the peptide amount was 1.5 pmol and 7.5 pmol. The uptake and internalization of [^177^Lu]Lu-DOTATOC dropped further by ~90% at the highest applied molar amount of peptide (75 pmol). The same pattern was observed in the case of [^177^Lu]Lu-DOTATOC-NLS. The uptake of [^177^Lu]Lu-DOTA-LM3 dropped by ~30% if 1.5 nmol of peptide were applied, similarly to [^177^Lu]Lu-DOTATOC and [^177^Lu]Lu-DOTATOC-NLS. On the other hand, the uptake declined by ~80% at the molar amount of 7.5 pmol, while under this condition the uptake of the other radiopeptides decreased by only 30%.

Experiments, in which excess of the non-labeled peptides was co-incubated to block the SSTR, demonstrated the drop in cell uptake of radiolabeled DOTATOC and DOTA-LM3 as previously published [11]. Specific uptake of [^177^Lu]Lu-DOTATOC-NLS was assessed by co-incubation of the AR42J tumor cells with 1 μM non-labeled DOTANOC and the radiopeptide. Under these conditions, the uptake of [^177^Lu]Lu-DOTATOC-NLS was below 0.2% of added activity.


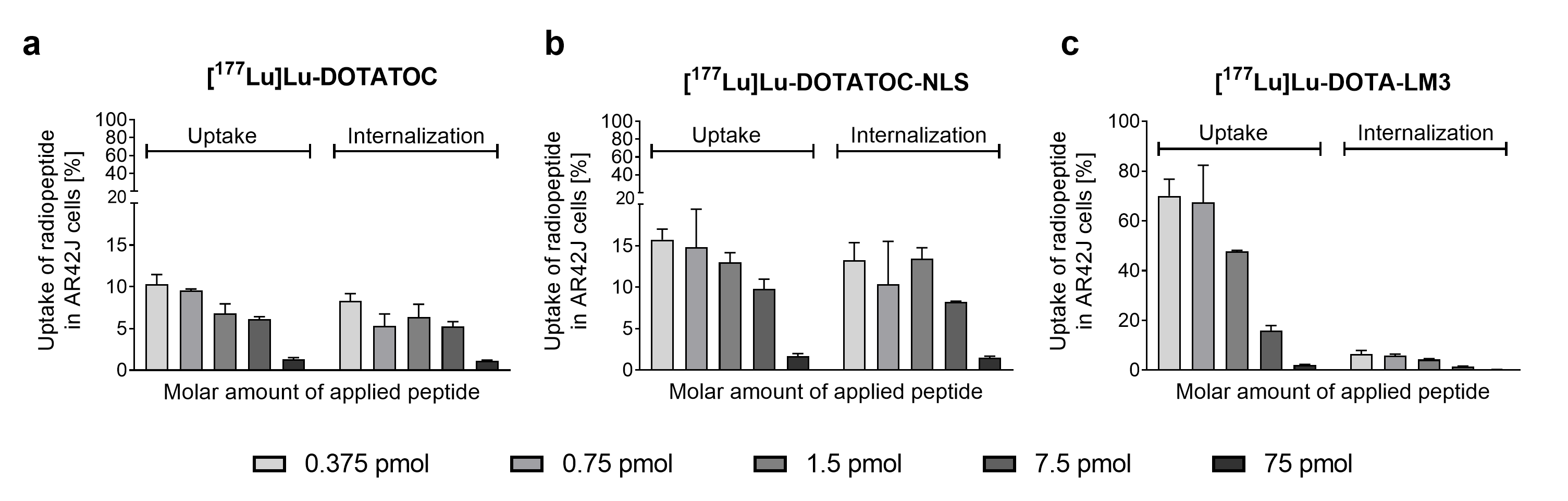


**Fig. S3** Results of the AR42J tumor cell uptake and internalization experiments performed with increasing molar amounts of non-labeled peptide. The radiopeptides were incubated with AR42J tumor cells for 2 h. (**a)** [^177^Lu]Lu-DOTATOC; (**b**) [^177^Lu]Lu-DOTATOC-NLS; (**c**) [^177^Lu]Lu-DOTA-LM3

**5. Nuclear localization of the radiopeptides**

***Purpose:*** The nucleus of AR42J tumor cells was isolated in order to determine the fraction of radiopeptides that localized in the cellular nucleus.

***Methods*:** AR42J tumor cells (10 x 10^6^) were seeded in PLL-coated Petri dishes using 15 mL cell culture medium with supplements and incubated overnight at 37 °C and 5% CO_2_. The next day, the medium was removed, the tumor cells were rinsed with PBS and 19.5 mL assay medium was added. The radiopeptides were added in a volume of 0.5 mL (2.5 MBq, 50 pmol) and the tumor cells incubated for 2 h at 37 °C. Afterwards, the cells were rinsed several times with PBS to remove the radiopeptides entirely. Subsequently, the cell nuclei and cytoplasm/membrane fractions were harvested according to the manufacturer’s protocol using the Nucli EZ Prep Nuclei Isolation Kit (Sigma Aldrich, USA). Ice-cold Nuclei EZ lysis buffer (4 mL) was added to each Petri dish to lyse the tumor cells followed by transfer of the respective cell suspension into an Eppendorf tube for centrifugation at 500 rcf for 5 min at 4 °C. The supernatant containing the cytoplasm/membrane fractions was transferred to a tube for counting the activity in a γ-counter. The pellet was resuspended in 4 mL ice-cold Nuclei EZ lysis buffer followed by additional centrifugation at 500 rcf for 5 min at 4 °C. The supernatant containing residual cytoplasm/membrane fractions was collected for counting the activity in a γ-counter. The pellet was resuspended in 200 µL Nuclei EZ storage buffer before transferring it into a tube for activity measurement in the γ-counter. The measured activity of the nuclei and cytoplasm/cell membrane fractions was defined as 100% of the cellular uptake. Nuclear localization was expressed as percentage of total cellular uptake. The collected fractions were stained with 0.4% trypan blue solution and analyzed using a microscope in order to confirm that the nuclei were properly separated from other cell fragments.

***Results*:** The results are reported in the main article.

**6. Cell viability assay**

***Purpose*:** Cell viability studies were performed using AR42J tumor cells to assess potential differences in the effects of ^161^Tb- and ^177^Lu-labeled DOTATOC, DOTATOC-NLS and DOTA-LM3.

***Methods*:** A total of 7500 AR42J tumor cells were seeded in 200 µL cell culture medium with supplements in PLL-coated 96-well plates. After incubation overnight at 37 °C and 5% CO_2_ to allow cell adhesion, the medium was removed and the cells were incubated with DOTATOC, DOTATOC-NLS or DOTA-LM3 radiolabeled with either terbium-161 or lutetium-177 at a molar activity of 100 MBq/nmol. The applied activity concentrations per well ranged between 0.001 MBq/mL and 40 MBq/mL (0.01–400 pmol/mL). After an incubation period of 2 h at 37 °C, the cells were rinsed once with PBS followed by addition of fresh cell culture medium with supplements. The tumor cells were allowed to grow for 6 d at 37 °C without changing cell culture medium and the cell viability was analyzed as previously described using a 3-(4,5-dimethylthiazol-2-yl)-2,5-diphenyltetrazolium bromide (MTT) assay [12]. After incubation of the cells with the MTT reagent for 2 h, the formed formazan crystals were dissolved in dimethyl sulfoxide. The absorption was measured at 560 nm with a microplate reader (560 nm, Victor^TM^ X3, Perkin Elmer, Waltham, MA, U.S.A.). The absorbance measured for untreated control cells was defined as 100% tumor cell viability. The viability of treated tumor cells (n=12 per concentration) was expressed as percentage of the absorbance of control cells. Data analysis and dose-response-fitting was carried out using GraphPad Prism software (version 8). The cell viability inhibition was calculated as the activity concentration which was necessary to reduce AR42J tumor cell viability to 50% of untreated control cells (EC_50_). EC_50_ values were determined in at least four independent experiments.

**Results:** The results are shown and discussed in the main article and Table S1.

**Table S1** Inhibition of the viability after exposure of AR42J tumor cells to the respective radiopeptide, expressed as half-maximum inhibitory concentration (EC_50_ values)

| **Radiopeptide** | **EC_50_ [MBq/mL]**  **(95% Confidence Interval)** | **Potency relative to**  **labeled DOTATOC** |
| --- | --- | --- |
| [^161^Tb]Tb-DOTATOC | 1.6  (1.4–1.9) | 1.0 |
| [^161^Tb]Tb-DOTATOC-NLS | 0.21  (0.17–0.26) | 7.8 |
| [^161^Tb]Tb-DOTA-LM3 | 0.010  (0.008–0.014) | 157 |
| [^177^Lu]Lu-DOTATOC | 8.2  (6.4–10) | 1.0 |
| [^177^Lu]Lu-DOTATOC-NLS | 0.88  (0.72–1.1) | 9.2 |
| [^177^Lu]Lu-DOTA-LM3 | 1.1  (0.8–1.5) | 7.7 |

**7. Cell survival assay**

**Purpose:** The capability of a single AR42J tumor cell to grow into a colony after exposure to ^161^Tb- and ^177^Lu-labeled DOTATOC, DOTATOC-NLS or DOTA-LM3 was determined by performing clonogenic assays [13].

**Methods:** In order to allow proper formation of colonies, 300 µL Matrigel (Growth Factor Reduced Basement Membrane Matrix, Corning Inc., New York, U.S.A; 2 mg/mL) diluted in RPMI cell medium without additives were added to each well of PLL-coated 6-well plates. AR42J tumor cells were seeded on the solidified Matrigel at a density of 2000 cells per well in 2 mL cell culture medium with supplements and incubated overnight at 37 °C and 5% CO_2_. The next day, the medium was removed and the cells were incubated with ^161^Tb- and ^177^Lu-labeled SST analogues (30 MBq/nmol) at activity concentrations of 0.01 MBq/mL to 0.5 MBq/mL (0.3–15 pmol/mL) for 2 h. The same procedure was applied to untreated control cells without exposure to radiopeptides. After incubation, the supernatant was discarded and the tumor cells were rinsed with PBS before fresh cell culture medium was added. After two weeks incubation time, the medium was removed and the cells were rinsed once with PBS. The colonies were stained using a crystal violet solution (0.5% crystal violet, 6% glutaraldehyde in water, 800 µL). The number of colonies (>0.1 mm) was determined visually using a grid of 0.5 cm x 0.5 cm in five selected squares under the microscope. The plating efficiency (PE) and survived fraction (SF) were calculated according to the following formulas: PE = ((number of colonies formed (untreated))/(number of cells seeded))*100; SF = ((number of colonies formed after treatment)/(number of cells seeded * PE))*100. The SF upon exposure to various radioactivity concentrations of the radioligands was determined in at least three independent experiments using triplicates in each experiment.

**Results:** The results of the cell survival determined by clonogenic assays are reported in the main manuscript.

**8. Determination of DNA DSBs in AR42J tumor cells after treatment**

**Purpose:** The study was performed to quantify the amount of double strand breaks (DSBs) in AR42J tumor cells treated with [^161^Tb]Tb-DOTATOC or [^177^Lu]Lu-DOTATOC, [^161^Tb]Tb-DOTATOC-NLS or [^177^Lu]Lu-DOTATOC-NLS as well as [^161^Tb]Tb-DOTA-LM3 or [^177^Lu]Lu-DOTA-LM3.

**Methods:** The number of DNA DSBs was assessed by immunostaining of γH2AX in AR42 tumor cells treated with either 2.5 MBq/mL or 10 MBq/mL of each radiopeptide. The cells were seeded (5 x 10^6^ cells/Petri dish) and let to grow overnight at 37 °C and 5% CO_2_. The following day, the medium was removed and the cells were treated with the radiopeptides diluted in assay medium for 2 h. The supernatant was then removed and the AR42J tumor cells rinsed with PBS prior to the addition of fresh medium. After 24 h of incubation, the cells were rinsed and detached by scraping with PBS followed by centrifugation. The tumor cell pellets were fixed with 10% neutral buffered formalin for 24 h at room temperature (RT) followed by exchanging it with PBS prior to paraffin embedding and sections preparation of 4 μm thickness. Briefly, after deparaffinization, antigen retrieval was performed with EDTA (pH 9) at 98 ºC for 20 min followed by incubation with REAL Antibody Diluent (Agilent Technologies, Santa Clara, California, USA) for 30 min at RT and hydrogen peroxide (Agilent Technologies, Santa Clara, California, USA) for 10 min at RT. The immunostaining was performed using a phospho-histone H2A.X (Ser139; dilution 1:200) rabbit monoclonal antibody (1 h at RT) (Cell Signaling Techonology, Danvers, Massachusetts,) and an anti-rabbit, horseradish peroxidase-derivatized secondary antibody (Agilent Technologies, Santa Clara, California, USA). Envision horseradish peroxidase rabbit detection system was used with DAB substrate buffer (Agilent Technologies, Santa Clara, California, USA). Immunostained sections were scanned using a digital slide scanner (NanoZoomer-XR C12000; Hamamatsu, Japan) and the total of positive and negative cells quantified with the pathology image analysis software VIS (Visiopharm Integrator System, Version 208 2019.02.2.6239, Visiopharm, Hoersholm, Denmark). First, the decision forest classification method was used to outline the tissue cell pellets as regions of interest (ROIs). Subsequently, the cell classification method was used for the detection of cell nuclei within each ROI and classify them as positive (brown) and negative (blue). Separation of the nucleus type was performed by training the software with the predetermined options “standard positive nuclei” and “standard negative nuclei”. The results were expressed as total positive cells and total negative cells.

**Results:** The results are reported in the main manuscript.

**9. SPECT/CT imaging studies**

***Purpose:*** SPECT/CT experiments were performed to investigate the time-dependent distribution of the radiolabeled DOTATOC, DOTATOC-NLS and DOTA-LM3 in AR42J tumor-bearing mice.

***Method:*** SPECT/CT scans were performed with a dedicated small-animal SPECT/CT scanner (NanoSPECT/CT, Mediso Medical Imaging Systems, Budapest, Hungary; Supplementary Material) [11]. The scans were acquired using Nucline software (version 1.02, Mediso Ltd., Budapest, Hungary) with energy windows at 47.7 keV (± 10%) and 74.6 keV (± 10%). SPECT data were reconstructed iteratively using HiSPECT software (version 1.4.3049, Scivis GmbH, Göttingen, Germany) [11]. The CT was reconstructed in real time using a cone-beam filtered backprojection. The fused datasets of SPECT and CT scans were analyzed using the VivoQuant postprocessing software (version 3.5, inviCRO Imaging Services and Software, Boston, USA). A Gauss post-reconstruction filter (full width at half maximum = 1.0 mm) was applied. Images were prepared using CorelDRAW (version X7).

The scans of AR42J tumor-bearing mice were performed when the tumor reached a volume of ~250 mm^3^. Mice were intravenously injected with [^161^Tb]Tb-DOTATOC, [^161^Tb]Tb-DOTATOC-NLS or [^161^Tb]Tb-DOTA-LM3 (~15 MBq, 1.0 nmol; in 100 µL PBS containing 0.05% bovine serum albumin (BSA) and ~150 μg ascorbic acid) and scanned 2 h, 4 h and 24 h afterwards. Blocking experiments were performed 2 h and 4 h after injection of the radiopeptides with an excess (20 nmol/mouse) of the respective unlabeled peptide. During the in vivo scans, the mice were anesthetized by inhalation of a mixture of isoflurane and oxygen.

***Results:*** The SPECT/CT images at 2 h p.i. of the radiopeptides are shown in the main article (Fig. 4). The scans obtained at 4 h and 24 h after injection of [^161^Tb]Tb-DOTATOC and [^161^Tb]Tb-DOTA-LM3 including the respective blocking studies were previously reported [11]. In brief, the uptake of [^161^Tb]Tb-DOTA-LM3 in the AR42J tumor xenografts was higher than for [^161^Tb]Tb-DOTATOC, whereas the uptake in the kidneys was comparable. This situation resulted in more favorable tumor-to-kidney ratios for [^161^Tb]Tb-DOTA-LM3 than for [^161^Tb]Tb-DOTATOC at all investigated timepoints. Moreover, SSTR-specific tumor uptake was confirmed by the fact that the tumor uptake was reduced to background levels in the presence of excess of the respective unlabeled peptide [11].

SPECT/CT scans of mice that received [^161^Tb]Tb-DOTATOC-NLS showed a significantly different distribution profile (Fig. S4). At all investigated timepoints the uptake of [^161^Tb]Tb-DOTATOC-NLS in the AR42J tumor xenografts was low, however, substantial accumulation of activity was visible in the liver and in the kidneys. While the activity was effectively cleared from the liver over time, the kidney retention of activity was still high at 24 h after injection of [^161^Tb]Tb-DOTATOC-NLS (Fig. S4a). Additional studies with mice that received excess of non-labeled DOTATOC-NLS blocked the tumor uptake effectively which indicated the SSTR-specific uptake of the radiopeptide (Fig. S4b). Liver uptake was reduced but not entirely blocked, however, retention in the kidneys remained unaffected irrespective of whether or not the non-labeled peptide was co-applied. These results indicated that the kidney uptake was not SSTR-related while the uptake mechanism in the liver may be specific to the DOTATOC-NLS’s structural features.


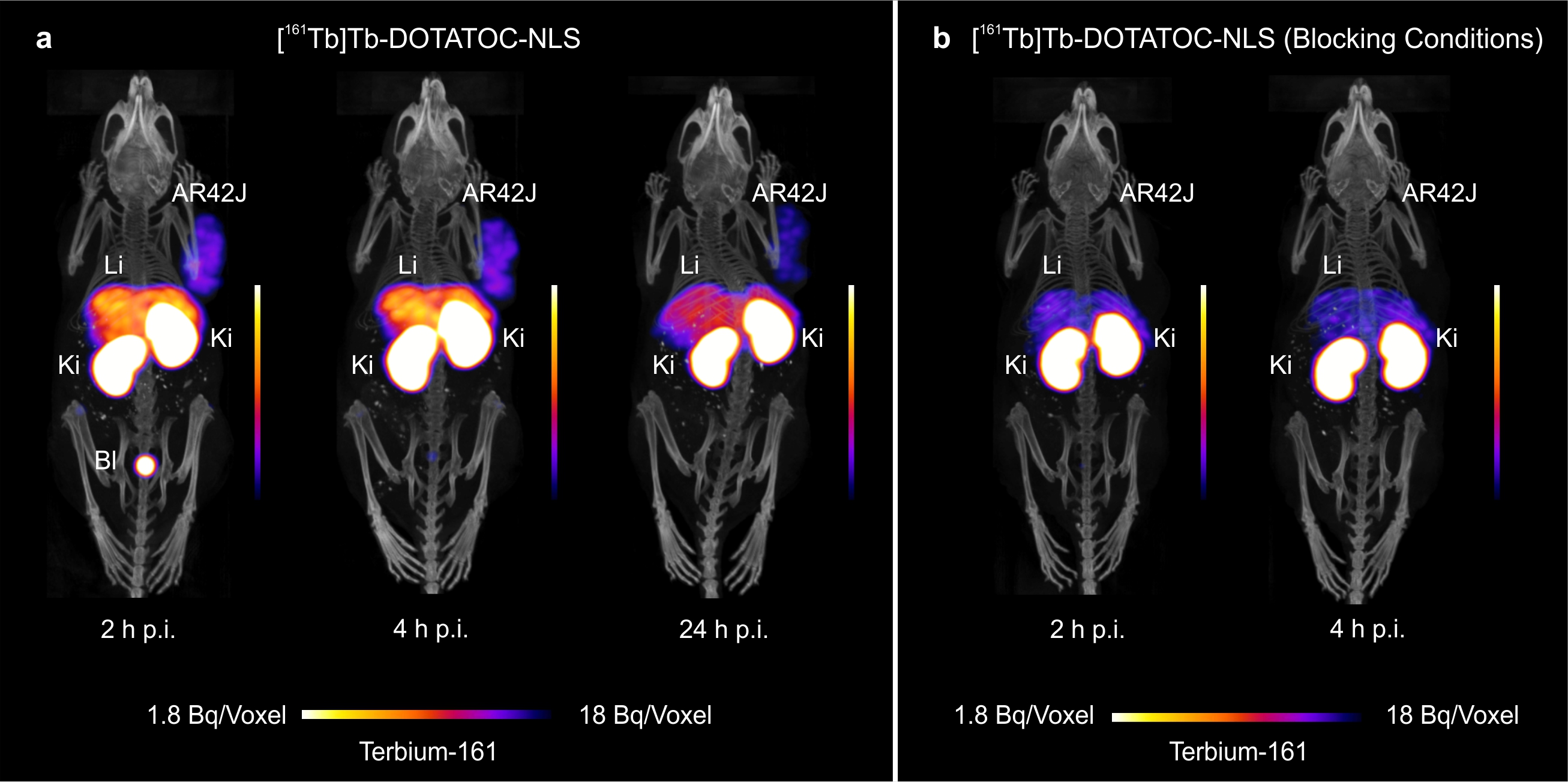


**Fig. S4.** SPECT/CT images of AR42J tumor-bearing mice shown as maximum intensity projections after injection of [^161^Tb]Tb-DOTATOC-NLS (15 MBq, 1.0 nmol per mouse). (**a**) Scans of mice at 2 h, 4 h and 24 h after injection of [^161^Tb]Tb-DOTATOC-NLS (**b**) Scans of mice at 2 h and 4 h after injection of [^161^Tb]Tb-DOTATOC-NLS and excess unlabeled DOTATOC-NLS. AR42J = SSTR-positive tumor xenograft; Ki = kidneys; Li = liver; Bl = urinary bladder

**10. Identification of the optimal molar amount of injected peptide**

***Purpose:*** The goal was to evaluate the effect of the injected amount of peptide on the biodistribution of ^161^Tb- and ^177^Lu-labeled DOTATOC and DOTA-LM3.

***Methods:*** Biodistribution studies were performed 10–14 days after tumor cell inoculation when the tumor size reached a volume of ~250 mm^3^. The mice (n=3 per group) were intravenously injected with 3–5 MBq radiolabeled DOTATOC or DOTA-LM3 (0.04 nmol/mouse, 0.2 nmol/mouse, or 1.0 nmol/mouse) in 100 μL PBS containing 0.05% BSA. The mice were sacrificed at 2 h p.i., selected tissues and organs were collected, weighed, and the accumulated activity was counted using a γ-counter. The decay-corrected data were listed as a percentage of the injected activity per gram of tissue mass (% IA/g).

***Results:*** As previously reported [14], the accumulation of activity in different organs and tissues was dependent on the molar amount of injected radiopeptide (Fig. S5, Tables S2 and S3). The tumor uptake of [^161^Tb]Tb-/[^177^Lu]Lu-DOTATOC applied at 0.04 nmol and 0.2 nmol per mouse was similar (~17% IA/g and ~15% IA/g, respectively, *p>*0.05), but significantly higher than after injection of 1.0 nmol per mouse (~8% IA/g; *p<*0.05). The uptake in the stomach was higher after injection of 0.04 nmol peptide compared to the injection of 0.2 nmol or 1.0 nmol peptide (*p<*0.05). The uptake in the pancreas, the adrenal glands and lungs showed a similar trend, but the difference among the different settings was not significant (*p>*0.05). No difference in uptake based on the amount of injected peptide was observed in the kidneys (~10%) and in the liver (~0.2% IA/g). [^161^Tb]Tb/[^177^Lu]Lu-DOTA-LM3 showed equally high tumor uptake after injection of 0.04 nmol and 0.20 nmol peptide per mouse (~34% and ~38% IA/g, *p>*0.05). The uptake was, however, reduced after injection of 1.0 nmol per mouse (~18% IA/g, *p<*0.05). Accumulation of the radiopeptide in the pancreas, adrenal glands, lungs and stomach was significantly higher after injection of 0.04 nmol peptide per mouse than after injection of 0.2 nmol per mouse (*p<*0.05). The lowest uptake was observed at an injected peptide amount of 1.0 nmol per mouse (*p<*0.05 compared to the injection of 0.2 and 1.0 nmol/mouse). The uptake in the liver was only slightly higher after injection of 0.04 nmol peptide per mouse (*p>*0.05) whereas the kidney uptake of [^161^Tb]Tb/[^177^Lu]Lu-DOTA-LM3 was in the range of 10% IA/g irrespective of the injected amount of peptide.


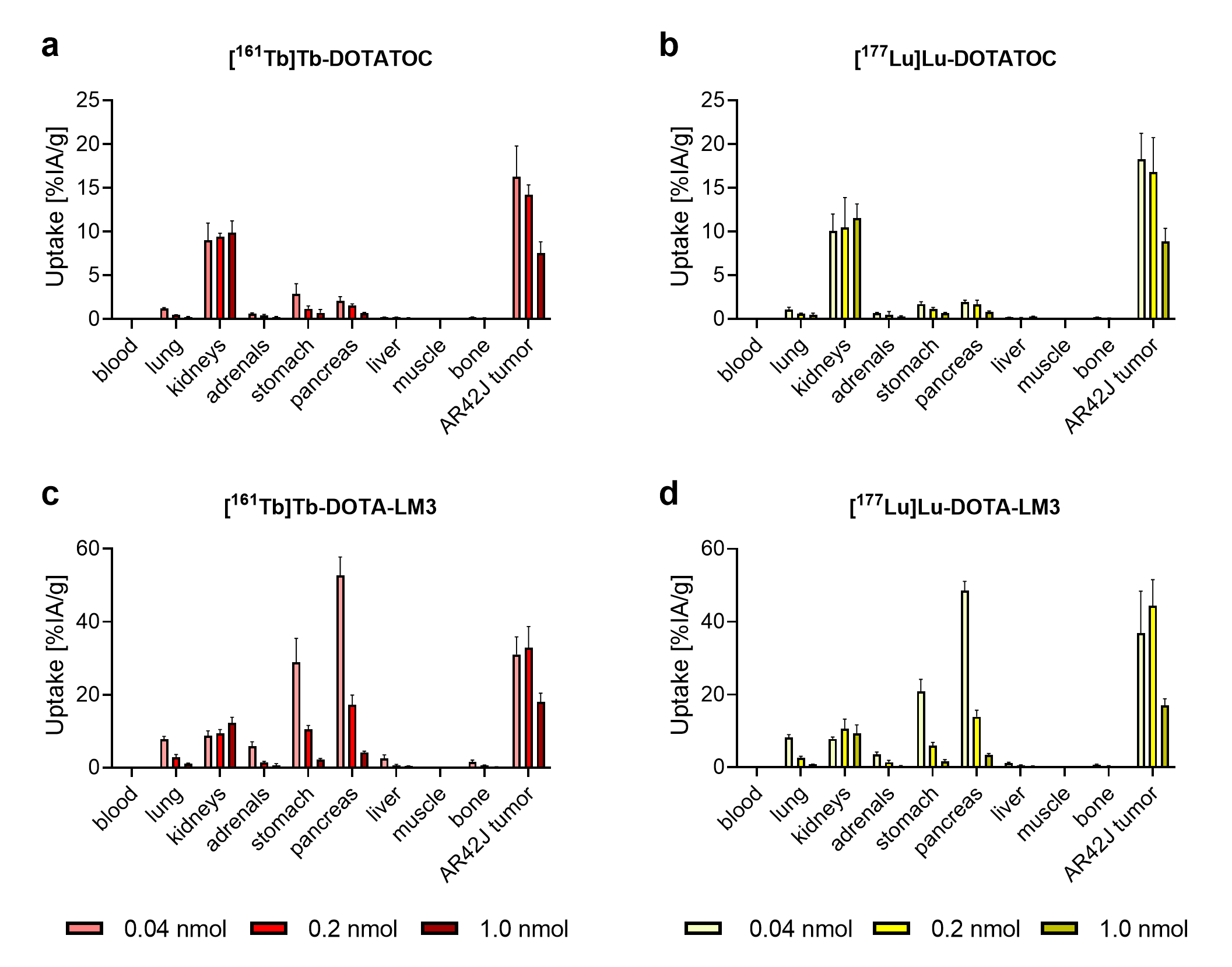
**Fig. S5** Biodistribution data obtained in AR42J tumor-bearing mice, 2 h after injection of the radiopeptides at different molar amounts. (**a**) Tissue distribution of [^161^Tb]Tb-DOTATOC; (**b**) Tissue distribution of [^177^Lu]Lu-DOTATOC; (**c**) Tissue distribution of [^161^Tb]Tb-DOTA-LM3; (**d**) Tissue distribution of [^177^Lu]Lu-DOTA-LM3. The results are presented as percentage of injected activity per gram tissue mass (% IA/g)

Consequently, the tumor-to-background ratios were strongly affected by the amount of injected radiopeptide (Tables S2 and S3). Tumor-to-kidney and tumor-to-liver ratios were favorable after injection of low molar amounts of peptide (0.04 nmol or 0.2 nmol) for both radiopeptides. The tumor-to-pancreas, tumor-to-adrenals, tumor-to-lungs and tumor-to-stomach ratios were higher after injection of 0.2 or 1.0 nmol in the case of DOTATOC than after injection of 0.04 nmol. These ratios were, however, more favorable after 1.0 nmol injected peptide of ^161^Tb- and ^177^Lu-labeled DOTA-LM3 than after using 0.2 nmol per mouse whereas the injection of only 0.04 nmol peptide per mouse resulted in the least favorable ratios.

Considering that the injection of 0.2 nmol resulted in the highest tumor uptake and, in the majority of the cases, in favorable tumor-to-background ratios, this molar amount of injected peptide was used for further in vivo studies.

**Table S2** Biodistribution data obtained in AR42J tumor-bearing mice at 2 h after injection of [^161^Tb]Tb-DOTATOC or [^177^Lu]Lu-DOTATOC. Decay-corrected data are shown as [% IA/g]-values, representing the average ± SD

| **Organ/Tissue** | **[^161^Tb]Tb-DOTATOC** | | | **[^177^Lu]Lu-DOTATOC** | | |
| --- | --- | --- | --- | --- | --- | --- |
|  | 2 h p.i. | | | 2 h p.i. | | |
|  | 0.040 nmol | 0.20 nmol | 1.0 nmol* | 0.040 nmol | 0.20 nmol | 1.0 nmol* |
| Blood | ≤0.10 | ≤0.10 | ≤0.10 | ≤0.10 | ≤0.10 | ≤0.10 |
| Heart | ≤0.10 | ≤0.10 | ≤0.10 | ≤0.10 | ≤0.10 | ≤0.10 |
| Lung | 1.2 ± 0.1 | 0.47 ± 0.05 | 0.27 ± 0.03 | 1.1 ± 0.2 | 0.63 ± 0.03 | 0.37 ± 0.06 |
| Spleen | 0.50 ± 0.11 | 0.18 ± 0.02 | 0.11 ± 0.02 | 0.39 ± 0.11 | 0.15 ± 0.02 | 0.15 ± 0.03 |
| Kidneys | 9.0 ± 2.0 | 9.4 ± 0.4 | 9.9 ± 1.3 | 10 ± 2 | 10 ± 3 | 11 ± 1 |
| Adrenals | 0.65 ± 0.08 | 0.43 ± 0.11 | 0.25 ± 0.06 | 0.65 ± 0.10 | 0.51 ± 0.35 | 0.28 ± 0.08 |
| Stomach | 2.9 ± 1.1 | 1.2 ± 0.3 | 0.74 ± 0.38 | 1.7 ± 0.3 | 1.1 ± 0.2 | 0.65 ± 0.11 |
| Pancreas | 2.1 ± 0.5 | 1.5 ± 0.2 | 0.71 ± 0.07 | 1.9 ± 0.2 | 1.7 ± 0.5 | 0.80 ± 0.13 |
| Intestines | 0.52 ± 0.15 | 0.24 ± 0.01 | 0.21 ± 0.13 | 0.33 ± 0.05 | 0.40 ± 0.25 | 0.20 ± 0.05 |
| Liver | 0.22 ± 0.02 | 0.19 ± 0.03 | 0.17 ± 0.01 | 0.18 ± 0.02 | 0.15 ± 0.03 | 0.26 ± 0.07 |
| Muscle | ≤0.10 | ≤0.10 | ≤0.10 | ≤0.10 | ≤0.10 | ≤0.10 |
| Femur | 0.23 ± 0.03 | 0.12 ± 0.03 | ≤0.10 | 0.17 ± 0.05 | 0.10 ± 0.01 | ≤0.10 |
| AR42J tumor | 16 ± 4 | 14 ± 1 | 8.2 ± 0.2 | 18 ± 3 | 17 ± 4 | 8.9 ± 1.5 |
| **Ratios** | 0.040 nmol | 0.20 nmol | 1.0 nmol* | 0.040 nmol | 0.20 nmol | 1.0 nmol* |
| Tu-to-blood | 187 ± 12 | 221 ± 56 | 260 ± 13 | 213 ± 41 | 235 ± 60 | 146 ± 16 |
| Tu-to-liver | 73 ± 3 | 76 ± 14 | 49 ± 1 | 100 ± 8 | 118 ± 24 | 36 ± 7 |
| Tu-to-kidney | 1.8 ± 0.2 | 1.5 ± 0.1 | 0.84 ± 0.12 | 1.9 ± 0.3 | 1.9 ± 0.4 | 0.73 ± 0.07 |
| Tu-to-panc | 7.8 ± 0.9 | 9.4 ± 1.3 | 12 ± 1 | 9.7 ± 1.8 | 10 ± 2 | 13 ± 1 |
| Tu-to-adr | 24 ± 1 | 34 ± 7 | 36 ± 10 | 28 ± 2 | 47 ± 29 | 29 ± 3 |
| Tu-to-lung | 13 ± 1 | 30 ± 4 | 33 ± 4 | 17 ± 3 | 27 ± 6 | 25 ± 3 |
| Tu-to-sto | 6 ± 2 | 12 ± 3 | 15 ± 2 | 11 ± 2 | 15 ± 4 | 15 ± 5 |

*Data reproduced from Borgna et al., 2021 [11]. (panc = pancreas, adr = adrenals, sto = stomach)

**Table S3** Biodistribution data obtained in AR42J tumor-bearing mice at 2 h after injection of [^161^Tb]Tb- DOTA-LM3 or [^177^Lu]Lu-DOTA-LM3. Decay-corrected data are shown as [% IA/g]-values, representing the average ± SD

| **Organ/Tissue** | **[^161^Tb]Tb-DOTA-LM3** | | | **[^177^Lu]Lu-DOTA-LM3** | | |
| --- | --- | --- | --- | --- | --- | --- |
|  | 2 h p.i. | | | 2 h p.i. | | |
|  | 0.040 nmol | 0.20 nmol | 1.0 nmol* | 0.040 nmol | 0.20 nmol | 1.0 nmol* |
| Blood | 0.12 ± 0.02 | ≤0.10 | ≤0.10 | 0.11 ± 0.01 | ≤0.10 | ≤0.10 |
| Heart | 0.34 ± 0.06 | 0.13 ± 0.01 | 0.11 ± 0.02 | 0.24 ± 0.04 | 0.14 ± 0.04 | ≤0.10 |
| Lung | 7.8 ± 0.7 | 2.9 ± 0.7 | 1.0 ± 0.2 | 8.2 ± 0.7 | 2.6 ± 0.4 | 0.83 ± 0.14 |
| Spleen | 1.8 ± 1.0 | 0.41 ± 0.08 | 0.27 ± 0.06 | 2.3 ± 1.9 | 0.41 ± 0.13 | 0.23 ± 0.06 |
| Kidneys | 8.8 ± 1.3 | 9.5 ± 1.0 | 12 ± 2 | 7.8 ± 0.5 | 11 ± 3 | 11 ± 1 |
| Adrenals | 5.9 ± 1.2 | 1.5 ± 0.2 | 0.44 ± 0.06 | 3.6 ± 0.6 | 1.4 ± 0.5 | 0.37 ± 0.10 |
| Stomach | 29 ± 7 | 11 ± 1 | 2.2 ± 0.3 | 21 ± 3.4 | 6.0 ± 0.9 | 1.7 ± 0.4 |
| Pancreas | 53 ± 5 | 17 ± 3 | 4.2 ± 0.3 | 49 ± 2.5 | 14 ± 2 | 3.4 ± 0.3 |
| Intestines | 2.0 ± 0.2 | 0.74 ± 0.11 | 0.43 ± 0.23 | 1.4 ± 0.6 | 0.53 ± 0.10 | 0.31 ± 0.14 |
| Liver | 2.6 ± 0.9 | 0.63 ± 0.24 | 0.45 ± 0.05 | 1.2 ± 0.2 | 0.51 ± 0.16 | 0.35 ± 0.08 |
| Muscle | ≤0.10 | ≤0.10 | ≤0.10 | ≤0.10 | ≤0.10 | ≤0.10 |
| Femur | 1.6 ± 0.5 | 0.53 ± 0.19 | 0.20 ± 0.05 | 0.75 ± 0.12 | 0.36 ± 0.05 | 0.17 ± 0.04 |
| AR42J tumor | 29 ± 6 | 33 ± 6 | 18 ± 2 | 37 ± 11 | 44 ± 7 | 17 ± 2 |
| **Ratios** | 0.040 nmol | 0.20 nmol | 1.0 nmol* | 0.040 nmol | 0.20 nmol | 1.0 nmol* |
| Tu-to-blood | 259 ± 22 | 461 ± 29 | 200 ± 38 | 289 ± 45 | 492 ± 102 | 270 ± 92 |
| Tu-to-liver | 13 ± 2 | 53 ± 9 | 38 ± 7 | 29 ± 8 | 90 ± 11 | 51 ± 7 |
| Tu-to-kidney | 3.5 ± 0.3 | 3.4 ± 0.1 | 1.4 ± 0.2 | 4.3 ± 1.2 | 4.3 ± 0.4 | 1.9 ± 0.2 |
| Tu-to-panc | 0.59 ± 0.05 | 1.9 ± 0.4 | 4.2 ± 0.4 | 0.77 ± 0.20 | 3.2 ± 0.3 | 4.2 ± 0.8 |
| Tu-to-adr | 5.4 ± 1.0 | 22 ± 5 | 36 ± 16 | 11 ± 4 | 34 ± 9.2 | 36 ± 6 |
| Tu-to-lung | 4.0 ± 0.6 | 12 ± 3 | 16 ± 4 | 4.3 ± 1.2 | 17 ± 2 | 18 ± 1 |
| Tu-to-sto | 1.1 ± 0.2 | 3.0 ± 0.3 | 8.5 ± 1.5 | 1.8 ± 0.6 | 7.4 ± 0.6 | 11 ± 2 |

*Data reproduced from Borgna et al., 2021 [11]. (panc = pancreas, adr = adrenals, sto = stomach)

**11. Time-dependent biodistribution studies**

***Purpose:*** Time-dependent biodistribution studies were performed to assess the total uptake of the radiopeptides in the AR42J tumor xenografts as well as in healthy organs and tissues.

***Methods:*** The methods are reported in the main article.

***Results:*** The biodistribution data of [^161^Tb]Tb-DOTATOC and [^161^Tb]Tb-DOTA-LM3 are shown as graphs in the main manuscript and listed below (Table S4/S5).

**Table S4** Biodistribution data obtained in AR42J tumor-bearing mice at 0.5 h, 2 h, 4 h, 24 h and 48 h after injection of 0.2 nmol of [^161^Tb]Tb-DOTATOC. Decay-corrected data are shown as [% IA/g]-values, representing the average ± SD

| **Organ/Tissue** | **[^161^Tb]Tb-DOTATOC (0.2 nmol/mouse)** | | | | |
| --- | --- | --- | --- | --- | --- |
|  | 0.5 h | 2 h | 4 h | 24 h | 48 h |
| Blood | 1.0 ± 0.1 | ≤0.10 | ≤0.10 | ≤0.10 | ≤0.10 |
| Heart | 0.45 ± 0.02 | ≤0.10 | ≤0.10 | ≤0.10 | ≤0.10 |
| Lung | 1.5 ± 0.1 | 0.47 ± 0.05 | 0.34 ± 0.06 | 0.15 ± 0.04 | 0.11 ± 0.02 |
| Spleen | 0.40 ± 0.09 | 0.18 ± 0.02 | 0.15 ± 0.00 | ≤0.10 | ≤0.10 |
| Kidneys | 11 ± 1 | 9.4 ± 0.4 | 11 ± 1 | 4.4 ± 0.9 | 1.8 ± 0.3 |
| Adrenals | 0.77 ± 0.18 | 0.43 ± 0.11 | 0.43 ± 0.03 | 0.16 ± 0.02 | 0.22 ± 0.07 |
| Stomach | 2.5 ± 0.3 | 1.2 ± 0.3 | 0.88 ± 0.09 | 0.78 ± 0.62 | 0.29 ± 0.05 |
| Pancreas | 3.8 ± 0.2 | 1.5 ± 0.2 | 1.1 ± 0.1 | 0.40 ± 0.02 | 0.23 ± 0.02 |
| Intestines | 0.47 ± 0.04 | 0.24 ± 0.01 | 0.20 ± 0.03 | 0.22 ± 0.25 | ≤0.10 |
| Liver | 0.41 ± 0.02 | 0.19 ± 0.03 | 0.15 ± 0.01 | ≤0.10 | ≤0.10 |
| Muscle | 0.24 ± 0.02 | ≤0.10 | ≤0.10 | ≤0.10 | ≤0.10 |
| Femur | 0.42 ± 0.04 | 0.12 ± 0.03 | ≤0.10 | ≤0.10 | ≤0.10 |
| AR42J tumor | 15 ± 1 | 14 ± 1 | 14 ± 1 | 6.3 ± 0.6 | 3.7 ± 0.7 |
| **Ratios** | 0.5 h | 2 h | 4 h | 24 h | 48 h |
| Tu-to-blood | 15 ± 2 | 221 ± 56 | 329 ± 17 | 490 ± 37 | 622 ± 14 |
| Tu-to-liver | 37 ± 2 | 76 ± 14 | 89 ± 3 | 67 ± 6 | 53 ± 3 |
| Tu-to-kidney | 1.4 ± 0.2 | 1.5 ± 0.1 | 1.3 ± 0.1 | 1.5 ± 0.2 | 2.1 ± 0.3 |

**Table S5** Biodistribution data obtained in AR42J tumor-bearing mice at 0.5 h, 2 h, 4 h, 24 h and 48 h after injection of 0.2 nmol of [^161^Tb]Tb-DOTA-LM3. Decay-corrected data are shown as [% IA/g]-values, representing the average ± SD

| **Organ/Tissue** | **[^161^Tb]Tb-DOTA-LM3 (0.2 nmol/mouse)** | | | | |
| --- | --- | --- | --- | --- | --- |
|  | 0.5 h | 2 h | 4 h | 24 h | 48 h |
| Blood | 1.3 ± 0.2 | ≤0.10 | ≤0.10 | ≤0.10 | ≤0.10 |
| Heart | 0.83 ± 0.25 | 0.13 ± 0.01 | ≤0.10 | ≤0.10 | ≤0.10 |
| Lung | 4.4 ± 1.4 | 2.9 ± 0.7 | 2.3 ± 0.4 | 0.94 ± 0.45 | 0.59 ± 0.11 |
| Spleen | 0.77 ± 0.09 | 0.41 ± 0.08 | 0.38 ± 0.02 | 0.18 ± 0.02 | 0.20 ± 0.01 |
| Kidneys | 12 ± 1 | 9.5 ± 1.0 | 9.0 ± 1.2 | 5.9 ± 0.8 | 3.8 ± 0.5 |
| Adrenals | 1.6 ± 0.4 | 1.5 ± 0.2 | 1.6 ± 0.6 | 0.73 ± 0.31 | 0.53 ± 0.08 |
| Stomach | 7.7 ± 2.1 | 11 ± 1 | 8.3 ± 0.3 | 3.8 ± 1.2 | 3.4 ± 0.5 |
| Pancreas | 15 ± 2 | 17 ± 3 | 16 ± 1 | 6.1 ± 0.7 | 3.7 ± 0.5 |
| Intestines | 1.0 ± 0.3 | 0.74 ± 0.11 | 0.81 ± 0.41 | 0.39 ± 0.21 | 0.25 ± 0.07 |
| Liver | 0.93 ± 0.23 | 0.63 ± 0.24 | 0.51 ± 0.08 | 0.29 ± 0.08 | 0.27 ± 0.04 |
| Muscle | 0.32 ± 0.03 | ≤0.10 | ≤0.10 | ≤0.10 | ≤0.10 |
| Femur | 0.92 ± 0.29 | 0.53 ± 0.19 | 0.54 ± 0.06 | 0.29 ± 0.15 | 0.24 ± 0.01 |
| AR42J tumor | 31 ±7 | 33 ± 6 | 35 ± 7 | 26 ± 4 | 21 ± 4 |
| **Ratios** | 0.5 h | 2 h | 4 h | 24 h | 48 h |
| Tu-to-blood | 24 ± 5 | 461 ± 29 | 820 ± 47 | 1371 ± 446 | 1249 ± 97 |
| Tu-to-liver | 34 ± 7 | 53 ± 9 | 69 ± 2 | 95 ± 30 | 78 ± 10 |
| Tu-to-kidney | 2.5 ± 0.4 | 3.4 ± 0.1 | 3.9 ± 0.1 | 4.5 ± 1.1 | 5.5 ± 0.8 |

12. Therapy study: scoring system and assessment of potential side effects

**Purpose:** The monitoring of the mice was performed to evaluate potential early side effects in mice treated with 2 x 10 MBq (0.2 nmol) of [^161^Tb]Tb-DOTATOC, [^177^Lu]Lu-DOTATOC, [^161^Tb]Tb-DOTA-LM3 or [^177^Lu]Lu-DOTA-LM3, respectively.

**Methods:**

***Definition of relative body weight (RBW):*** The RBW was defined as [BW_x_/ BW_0_], where BW_x_ is the body weight in gram at a given Day x and BW_0_ the body weight in gram at Day 0.

***Definition of tumor volume (V) and relative tumor volume (RTV):*** The tumor dimension was determined by measuring the longest tumor axis (L) and its perpendicular axis (W) with a digital caliper. The tumor volume (TV) was calculated according to the equation [V = 0.5 × (L × W^2^)]. The relative tumor volume (RTV) was defined as [TV_x_/TV_0_], where TV_x_ is the tumor volume in mm^3^ at a given Day x and TV_0_ the tumor volume in mm^3^ at Day 0.

***Scoring system:*** Endpoint criteria were defined using a scoring system which required euthanasia of mice with a score ≥3. Every second day, the following criteria were assessed in the mice, assigning a score from 0–3 for each criterion: (i) appearance (general status, skin color, etc.), (ii) behavior (vitality, sociality, crouching etc.), (iii) body weight (stable, loss >5 ≤10%, loss >10 <15%, loss ≥ 15% compared to initial body weight), (iv) tumor size (<800 mm^3^, ≥800 and <900 mm^3^, ≥900 and <1000 mm^3^, ≥1000 mm^3^), (v) tumor ulceration. A score ≥3 was for example due to: (i) appearance of wrinkled, translucent skin, (ii) mouse in crouching position and/or apathetic, (iii) a body weight loss of ≥15% of initial body weight, (iv) a tumor volume of ≥1000 mm^3^, (iii/iv) a combination of a tumor size of ≥800 mm^3^ and body weight loss of ≥10% and/or (v) ulceration of the tumor.

***Blood plasma chemistry:*** Immediately before euthanasia of the mice that had reached the endpoint, blood was sampled from the retrobulbar vein. The values of creatinine (CRE), blood urea nitrogen (BUN), alkaline phosphatase (ALP), total bilirubin (TBIL) and albumin (ALB) were determined in the blood plasma, after centrifugation of the blood, using a dry chemistry analyzer (DRI-CHEM 4000i, FUJIFILM, Japan). The average blood plasma parameters of each group of mice were analyzed for significance using a one-way ANOVA test with a Tukey’s multiple comparisons post-test (GraphPad Prism software, version 8). A *p*-value of <0.05 was considered as statistically significant.

***Organ mass and mass ratios at the endpoint:*** Mice were euthanized when a predefined endpoint criterion was reached or when the study was terminated at Day 49. Organ masses, organ mass ratios (kidney-to-brain, liver-to-brain and spleen-to-brain) and organ mass-to-body weight ratios (kidney-to-body, liver-to-body and spleen-to-body) were analyzed for significance using a one-way ANOVA test with a Tukey’s multiple comparisons post-test (GraphPad Prism software, version 8). A *p*-value of <0.05 was considered as statistically significant.

**Results:**

***Relative body weight, organ mass and mass ratios:*** The relative body weights of treated mice measured at Day 6 and at the endpoint were comparable among the groups (*p>*0.05) pointing at a general well-being of the mice and the absence of early side effects due to the treatment (Table S6). In addition, organ masses and mass ratios were comparable among the groups at the endpoint (*p>*0.05), Tables S7/S8.

**Table S6** Relative body weights of mice at Day 6 and at the endpoint of the therapy study. No significant difference among the groups was observed (*p>*0.05)

| **Group**  (n=6) | **Relative body weight at Day 6^1^**  (average ± SD) | **Relative body weight at endpoint^2^**  (average ± SD) |
| --- | --- | --- |
| A | 1.03 ± 0.03 | 1.05 ± 0.03 |
| B | 1.02 ± 0.03 | 1.07 ± 0.02 |
| C | 0.99 ± 0.03 | 1.01 ± 0.07 |
| D | 1.04 ± 0.03 | 1.06 ± 0.07 |
| E | 1.01 ± 0.02 | 1.11 ± 0.06 |

^1^ Data obtained at Day 6 when the first control mouse reached an endpoint.

^2^ Data obtained at the day of euthanasia when an endpoint criterion was reached or at the end of the study.

**Table S7** Organ mass of mice of the therapy study collected after euthanasia. No significant difference among the groups was observed (*p>*0.05)

| **Group**  (n=6) | **Organ mass**^1^ (mg)  (average ± SD) | | | |
| --- | --- | --- | --- | --- |
|  | Kidneys | Liver | Spleen | Brain |
| A | 327 ± 28 | 1096 ± 128 | 90 ± 10 | 429 ± 23 |
| B | 352 ± 27 | 1195 ± 57 | 86 ± 19 | 440 ± 23 |
| C | 342 ± 23 | 1121 ± 123 | 90 ± 17 | 453 ± 31 |
| D | 332 ± 46 | 1275 ± 254 | 85 ± 27 | 465 ± 30 |
| E | 336 ± 13 | 1254 ± 106 | 92 ± 17 | 471 ± 30 |

^1^ Data obtained at the day of euthanasia when an endpoint criterion was reached or at the end of the study.

**Table S8** Organ mass-to-brain mass and organ mass-to-body weight ratios. No significant difference among the groups was observed (*p>*0.05)

| **Group**  (n=6) | **Organ mass-to-brain mass ratios**  (average ± SD) | | |
| --- | --- | --- | --- |
|  | Kidney-to-brain | Liver-to-brain | Spleen-to-brain |
| A | 0.77 ± 0.09 | 2.6 ± 0.3 | 0.21 ± 0.03 |
| B | 0.80 ± 0.03 | 2.7 ± 0.2 | 0.20 ± 0.05 |
| C | 0.76 ± 0.03 | 2.5 ± 0.2 | 0.20 ± 0.03 |
| D | 0.72 ± 0.12 | 2.8 ± 0.7 | 0.19 ± 0.07 |
| E | 0.72 ± 0.05 | 2.7 ± 0.1 | 0.20 ± 0.04 |
| **Group**  (n=6) | **Organ mass-to-body weight ratios**  (average ± SD) | | |
|  | Kidney-to-body | Liver-to-body | Spleen-to-body |
| A | 0.013 ± 0.001 | 0.047 ± 0.002 | 0.004 ± 0.000 |
| B | 0.013 ± 0.001 | 0.049 ± 0.004 | 0.003 ± 0.001 |
| C | 0.014 ± 0.001 | 0.050 ± 0.003 | 0.004 ± 0.001 |
| D | 0.014 ± 0.001 | 0.050 ± 0.001 | 0.004 ± 0.001 |
| E | 0.014 ± 0.001 | 0.050 ± 0.003 | 0.003 ± 0.001 |

*Blood plasma chemistry:* No significant differences in blood plasma parameters were observed among the groups with the exception of BUN. In this case, Groups D and E, which received [^161^Tb]Tb-DOTA-LM3 and [^177^Lu]Lu-DOTA-LM3, respectively, had significantly elevated values (*p<*0.05) (Table S9). Nevertheless, the values were in the physiological range for mice based on listed values of the respective breeding company (Charles River, Sulzfeld, Germany).

Table S9 Plasma chemistry determined at the endpoint of the therapy (n=6, if not otherwise indicated)

| Group | **ALB** | **CRE** | **BUN** | **ALP** | **TBIL** |
| --- | --- | --- | --- | --- | --- |
|  | (g/L) | (µmol/L) | (mmol/L) | (U/L) | (µmol/L) |
| A | 22 ± 2 | <18 (n=6) | 6.2 ± 0.7 | 71 ± 15 | <3 (n=2)  3 ± 1 (n=4) |
| B | 23 ± 2 | <18 (n=6) | 5.8 ± 1.0 | 76 ± 21 | <3 (n=4)  4 ± 1 (n=2) |
| C | 24 ± 5 | <18 (n=5) | 6.3 ± 0.9 | 77 ± 8 | <3 (n=3)  4 ± 1 (n=3) |
| D | 22 ± 1 | <18 (n=5)  18 (n=1) | 9.1 ± 1.5* | 65 ± 15 | <3 (n=5)  3 (n=1) |
| E | 23 ± 1 | <18 (n=5)  19 (n=1) | 8.2 ± 1.8** | 66 ± 5 | <3 (n=4)  4 ± 1 (n=2) |

* Significantly different (*p<*0.05) from the values of Groups A, B, C.

** Significantly different (*p<*0.05) from the value of Group B.

13. Terbium-161 production: current status and future development

**Purpose:** In view of a clinical application of [^161^Tb]Tb-DOTA-LM3 and, possibly, other ^161^Tb-based radiopharmaceuticals in future, the availability of n.c.a. terbium-161 in sufficient activity and at high quality is crucial. Therefore, current endeavors in our laboratories at PSI, and in collaboration with others, are focused on the scale-up process of the terbium-161 production.

**Current production process of terbium-161:** Terbium-161 is currently produced via the ^160^Gd(n,γ)^161^Gd→^161^Tb nuclear reaction (Fig. S6) [1], in analogy to the production of n.c.a. lutetium-177, which is obtained via the ^176^Yb(n,γ)^177^Yb→^177^Lu nuclear reaction and is commercially available from different suppliers. Enriched gadolinium-160 targets are irradiated with neutrons at a high-flux reactor in Pelindaba, South Africa, or in Grenoble, France, as previously reported by Gracheva et al. [2]. It is also feasible to use other high flux reactors or the spallation neutron source at PSI (SINQ) for target irradiation. The separation of terbium-161 from the gadolinium target material has been developed and improved at PSI over the last 10 years (Fig. S6). This process is based on ion exchange chromatographic methods, as is the case for lutetium-177. The terbium-161 produced is finally eluted in dilute hydrochloric acid (HCl 0.05 M) with comparable specifications to those provided for commercial lutetium-177 [2].

**Availability of terbium-161 in future – potential scenarios:** Any reactor facility suitable to irradiate ytterbium-176 targets for the production of lutetium-177 can potentially be used for the irradiation of gadolinium-160 targets to produce terbium-161. Until recently, terbium-161 was not listed in the dangerous goods tables of the ADR (European Agreement concerning the International Carriage of Dangerous Goods by Road) and International Air Transport Agency (IATA) regulations. The generic A2 value (type A activity limit of β¯-particle emitter or γ-ray emitter) of 0.02 TBq had to be applied for the transport of irradiated target material (“Basic Radionuclide Values for Unknown Radionuclides or Mixtures”). This restriction has been lifted in 2021. The dedicated A2 value of terbium-161 has been defined as 0.7 TBq, identical to that of lutetium. This will enables the shipping of higher activities of irradiated material and, hence, the regular production of higher batch activities of terbium-161.

Any subsequent step, including the preparation of the radiopeptides under GMP conditions using an automated synthesis module, as well as the distribution of the formulated radiopharmaceutical, can be performed in analogy to the production of ^177^Lu-labeled peptides for clinical application (Fig. S6).

To provide scientific evidence of the advantages of using terbium-161 for therapeutic purposes is of critical importance and, thus, the first step needed to be taken in order to attract the attention of industrial partners that would be interested in making terbium-161 available for clinicians worldwide.


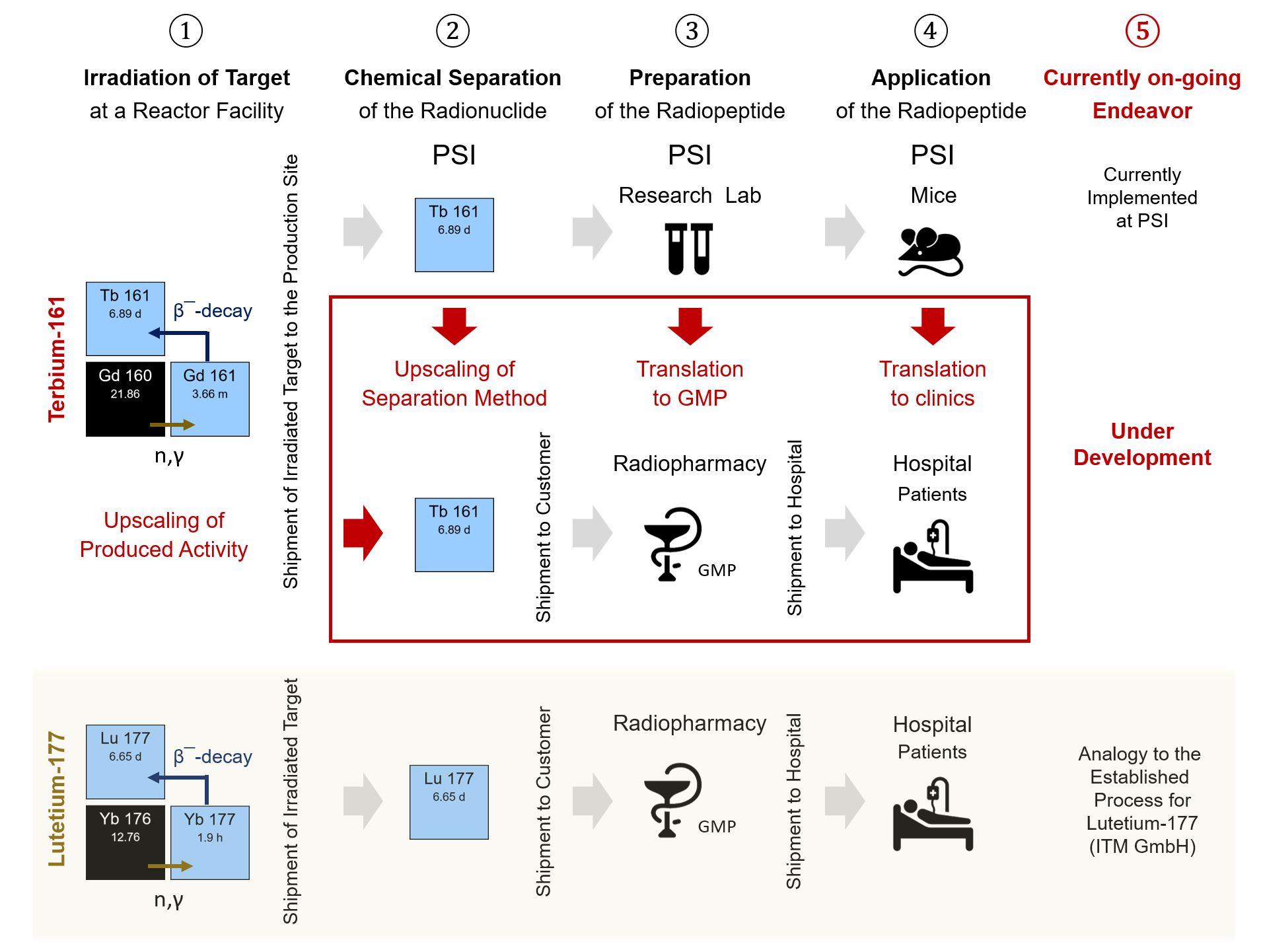


**Fig. S6** Sketch of the current production and use of n.c.a. terbium-161 at Paul Scherrer Institute, Switzerland (upper panel), the scale-up process which is currently under development (middle panel) and the analogy to the established production and application of n.c.a. lutetium-177 that is already commercially available.

**References**

1. Lehenberger S, Barkhausen C, Cohrs S, Fischer E, Grünberg J, Hohn A, et al. The low-energy beta^-^ and electron emitter ^161^Tb as an alternative to ^177^Lu for targeted radionuclide therapy. Nucl Med Biol. 2011;38:917-24. doi:S0969-8051(11)00044-8 [pii] 10.1016/j.nucmedbio.2011.02.007.

2. Gracheva N, Müller C, Talip Z, Heinitz S, Köster U, Zeevaart JR, et al. Production and characterization of no-carrier-added ^161^Tb as an alternative to the clinically-applied ^177^Lu for radionuclide therapy. EJNMMI Radiopharm Chem. 2019;4:12. doi:10.1186/s41181-019-0063-6.

3. Ginj M, Mäcke HR. Synthesis of trifunctional somatostatin based derivatives for improved cellular and subcellular uptake. Tetrahedron Lett. 2005;46:2821-4. doi:10.1016/j.tetlet.2005.02.117.

4. Ginj M, Hinni K, Tschumi S, Schulz S, Mäcke HR. Trifunctional somatostatin-based derivatives designed for targeted radiotherapy using auger electron emitters. J Nucl Med. 2005;46:2097-103.

5. Fani M, Del Pozzo L, Abiraj K, Mansi R, Tamma ML, Cescato R, et al. PET of somatostatin receptor-positive tumors using ^64^Cu- and ^68^Ga-somatostatin antagonists: the chelate makes the difference. J Nucl Med. 2011;52:1110-8. doi:10.2967/jnumed.111.087999.

6. Baum RP, Kluge AW, Kulkarni H, Schorr-Neufing U, Niepsch K, Bitterlich N, et al. [^177^Lu-DOTA]^0^-D-Phe^1^-Tyr^3^-Octreotide (^177^Lu-DOTATOC) for peptide receptor radiotherapy in patients with advanced neuroendocrine tumours: a Phase-II study. Theranostics. 2016;6:501-10. doi:10.7150/thno.13702.

7. Wang LF, Lin L, Wang MJ, Li Y. The therapeutic efficacy of ^177^Lu-DOTATATE/DOTATOC in advanced neuroendocrine tumors: a meta-analysis. Medicine (Baltimore). 2020;99:e19304. doi:10.1097/MD.0000000000019304.

8. Fani M, Braun F, Waser B, Beetschen K, Cescato R, Erchegyi J, et al. Unexpected sensitivity of sst2 antagonists to N-terminal radiometal modifications. J Nucl Med. 2012;53:1481-9. doi:10.2967/jnumed.112.102764.

9. Müller C, Reber J, Haller S, Dorrer H, Bernhardt P, Zhernosekov K, et al. Direct in vitro and in vivo comparison of ^161^Tb and ^177^Lu using a tumour-targeting folate conjugate. Eur J Nucl Med Mol Imaging. 2014;41:476-85. doi:10.1007/s00259-013-2563-z.

10. Müller C, Umbricht CA, Gracheva N, Tschan VJ, Pellegrini G, Bernhardt P, et al. Terbium-161 for PSMA-targeted radionuclide therapy of prostate cancer. Eur J Nucl Med Mol Imaging. 2019;46:1919-30. doi:10.1007/s00259-019-04345-0.

11. Borgna F, Barritt P, Grundler PV, Talip Z, Cohrs S, Zeevaart JR, et al. Simultaneous visualization of ^161^Tb- and ^177^Lu-labeled somatostatin analogues using dual-isotope SPECT imaging. Pharmaceutics. 2021;13. doi:10.3390/pharmaceutics13040536.

12. Mosmann T. Rapid colorimetric assay for cellular growth and survival: application to proliferation and cytotoxicity assays. J Immunol Methods. 1983;65:55-63. doi:10.1016/0022-1759(83)90303-4.

13. Franken NA, Rodermond HM, Stap J, Haveman J, van Bree C. Clonogenic assay of cells in vitro. Nat Protoc. 2006;1:2315-9. doi:10.1038/nprot.2006.339.

14. Nicolas GP, Mansi R, McDougall L, Kaufmann J, Bouterfa H, Wild D, et al. Biodistribution, pharmacokinetics, and dosimetry of ^177^Lu-, ^90^Y-, and ^111^In-labeled somatostatin receptor antagonist OPS201 in comparison to the agonist ^177^Lu-DOTATATE: the mass effect. J Nucl Med. 2017;58:1435-41. doi:10.2967/jnumed.117.191684.
